# Antibacterial Effects of *Commiphora gileadensis* Methanolic Extract on Wound Healing

**DOI:** 10.3390/molecules27103320

**Published:** 2022-05-21

**Authors:** Ayman Alhazmi, Abdullah F. Aldairi, Ahmad Alghamdi, Anas Alomery, Abdulrahman Mujalli, Ahmad A. Obaid, Wesam F. Farrash, Mamdouh Allahyani, Ibrahim Halawani, Abdulelah Aljuaid, Sarah A. Alharbi, Mazen Almehmadi, Moodi S. Alharbi, Anmar A. Khan, Maisam A. Jastaniah, Abdulrhman Alghamdi

**Affiliations:** 1 Department of Clinical Laboratories Sciences, College of Applied Medical Sciences, Taif University, P.O. Box 11099, Taif 21944, Saudi Arabia; aboteef999@hotmail.com (A.A.); a.ghamadi@tu.edu.sa (A.A.); omarianas1@hotmail.com (A.A.); m.allahyani@tu.edu.sa (M.A.); i.halawani@tu.edu.sa (I.H.); ab.aljuaid@tu.edu.sa (A.A.); dr.mazen.ma@gmail.com (M.A.); 2 Laboratory Medicine Department, Faculty of Applied Medical Sciences, Umm Al-Qura University, Al Abdeyah, P.O. Box 7607, Makkah 21961, Saudi Arabia; ammujalli@uqu.edu.sa (A.M.); aaobaid@uqu.edu.sa (A.A.O.); wffarrash@uqu.edu.sa (W.F.F.); aaakhan@uqu.edu.sa (A.A.K.); 3 Laboratory Department, Prince Mohammed Bin Abdulaziz Hospital, Ministry of National Guard-Health Affairs, Al Madinah 41511, Saudi Arabia; alharbisara2121@gmail.com; 4 Diabetic Centre, King Abdulaziz Speciality Hospital, Ministry of Health, Qurwa, Taif 26521, Saudi Arabia; moodi-1411@hotmail.com; 5 Laboratory Department, King Faisal Hospital, Ministry of Health, Makkah 24236, Saudi Arabia; mjastauiah@moh.gov.sa; 6 Faculty of Medicine, Taif University, Taif 21944, Saudi Arabia; mzamt@hotmail.com

**Keywords:** *Commiphora gileadensis*, skin wound healing, wound contraction, *Staphylococcus aureus*

## Abstract

*Commiphora gileadensis* (*CG*) is a small tree distributed throughout the Middle East. It was traditionally used in perfumes in countries in this area. In Saudi Arabia, it was used to treat wounds burns and as an antidote to scorpion stings. This study aimed to evaluate the antimicrobial activity and cutaneous wound healing efficiency of the *CG* extracts using microbiological tests, rate of wound contraction and histopathological changes. *CG* plant were extracted using the methanol extraction technique; then, the methanolic extract was characterized using liquid chromatography coupled with mass spectrometry (LC–MS). Afterwards, a six-millimetre (mm) excision wound was induced in 60 male Balb/c mice. Mice were classified into two classes; each class consisted of three groups of 10 mice. In the non-infected wound class, the group I was assigned as control and received normal saline. Group II received gentamicin treatment, and group III treated with *CG*-methanolic extract. In the *Staphylococcus aureus*-infected class, group IV received normal saline, and groups V and VI were treated with gentamicin and *CG*-methanolic extract, respectively. The colonization of infected wounds was determined using colony-forming units (CFUs), and the percentage of wound contraction was measured in all groups. Finally, the histopathologic semi-quantitative determination of wound healing was evaluated by inflammatory cell infiltration, the presence of collagen fibres and granulation tissue, and the grade of re-epithelization. Composition analysis of the methanolic extract confirmed the presence of a high amount of ceramide (69%) and, to a lesser extent, hexosylceramide (18%) and phosphatidylethanolamine (7%) of the total amount. Additionally, there was a statistically significant difference between the percentage of wound contraction in the *CG*-treated and control groups in both *Staphylococcus aureus*-infected and non-infected wounds (*p* < 0.01). The colonization of the infected wounds was lower in the group treated with *CG* than in the control group (*p* < 0.01). In both non-infected and infected wounds, the *CG*-treated group showed significant statistical differences in inflammatory cell infiltration, collagen fibres, re-epithelization and granulation tissue formation compared with the control group (*p* < 0.01). The *CG* extract possesses antibacterial and anti-inflammatory properties that induce wound healing.

## 1. Introduction

In the human body, the skin is the first line of defence mechanism that protects the body from the external environments [1], where significant skin damage may lead to several complications and death, for instance, wounds in diabetic patients [2,3]. Wounds can be classed based on a variety of factors. In the treatment of injuries and wounds, time is crucial. According to the length of time it takes for a wound to heal, it can be classified as acute or chronic [4]. In contrast, acute wounds can be defined as wounds that self-heal and heal normally, with both functional and anatomical restoration as a result of following a timely and ordered healing process [3,5]. On the other hand, chronic wounds do not heal correctly or in a timely manner and do not move through the regular stages of healing [6]. Consequently, after a skin injury, the body initiates a physiological response known as the normal wound healing process [7]. The reaction involves different functions, including haemostasis, inflammation, cell proliferation and maturation, and remodeling [8,9], mediated through several cytokines, chemical mediators, and secretions from various cell types [10,11].

The first stage starts when keratinocytes at the skin breach site produce interleukin-1 (IL-1) and tumour necrosis factor-α (TNF-α). These cytokines stimulate adjacent cells to reduce the wound area [12]. Moreover, the disruption of blood vessels at the injury site induces platelet aggregation and activates blood coagulation and complement cascades [13,14,15]. Inflammatory cells are then get involved in the site of injury via platelets released growth factors such as epidermal growth factor (EGF), platelet-derived growth factor (PDGF) and transforming growth factor-β (TGF-β) [16]. Neutrophils are recognized to be the first inflammatory cells that reach the site of injury to remove damaged cells, bacteria, amongst other foreign materials [17], which are usually followed by the proliferation of the epidermis’ basal layer at the edge of the incision [18]. Different cell types, such as keratinocytes and monocytes, migrate to the injury site and proliferate in the second stage. Monocytes proliferate into macrophages under the influence of TGF-β, which initiates tissue granulation to fill the injury site [19]. The angiogenesis is maximized by the fifth day of wound healing and by the end of stage two, the fibroblasts differentiate into contractile myofibroblasts [20,21]. In the third stage, fibroblasts and myofibroblasts produce collagen and extracellular matrix components that bridge the wound edges. Wound remodeling, the last step, begins two weeks after the epidermis abrasion and lasts for a year [17].

Chronic wounds could not be healed quickly for various reasons; for instance, infection is one of the leading reasons for delayed wound healing; hence, infection control should be considered a top priority in wound care. Wounds should be treated with an aseptic approach, adequate debridement, and suitable antimicrobial medications [22]. A concentrated topical antimicrobial agent is considered effective in wound management, decreasing systemic side effects and antimicrobial resistance [23]. Broad-spectrum antibiotics are less toxic; however, antibacterial resistance is significant. Indeed, using natural products with antimicrobial effects would enhance the impact of microbial elimination in the healing process [24]. *Commiphora gileadensis* (*CG*) belongs to the Burseraceae family, grows in Saudi Arabia and is traditionally used to treat different diseases [25,26]. CG plant bark was recommended as an anti-hypertensive, anti-inflammatory, and pain killer for fever and pain symptoms [27,28,29]. It remains an important medicinal plant and is utilized to treat pain and fever and skin infections [30]. A previous study showed that the *CG* methanolic extract had an antibacterial effect on *methicillin-resistant Staphylococcus aureus* (MRSA) and *Pseudomonas aeruginosa* [31,32,33]. This study aimed to assess the effect of *CG* methanolic extract on the healing of wounds infected with *Staphylococcus aureus*.

## 2. Materials and Methods

### 2.1. CG Collection

Parts from the *CG* tree were collected from a high mountain area called the Alaab Valley (24°05′09.5″ N, 38°58′31.8″ E), the western area of the Makkah region, Saudi Arabia. Leaves and fallen branches were collected during July 2020.

### 2.2. Preparation of CG Methanolic Extract

Leaves and branches were cleaned with water and then dried using a vacuum oven (Sheldon^®^, Grand Rapids, MI, USA) at 40 °C under 50 mmHg for 8 h. Then, leaves were ground into a fine powder using razor blade to remove any large particles [34]. A 10 g portion of the powder was then macerated in 100 mL of methanol in a sterile container and left for 24 h. Afterwards, the container was vigorously shaken, and the extract was filtered using 0.22 µm filter paper (Millipore^®^, Burlington, MA, USA); then, the extract was dried at 40 °C using a rotary evaporator (Buchi, Essen, Germany). Finally, the extract was stored at −20 °C for further analysis.

### 2.3. Sample Characterization Using Ultraperformance Liquid Chromatography Coupled with Mass Spectrometer (UPLC–MS)

Samples were thawed on ice and added with 1.5 mL of Chloroform:Methanol (2:1, *v*/*v*), 0.5 mL ultrapure water into the sample, vortexed for 1 min, centrifuge 10 min at 3000 rpm at 4 °C. Transfer the lower phase to a new tube, dry under the nitrogen. Then the dried extract was resuspended with 200 μL of isopropyl alcohol: MeOH (1:1, *v*/*v*); add 5 μL of 1-heptadecanoyl-2-hydroxy-sn-glycero-3-phosphocholine LPC (12:0) as internal standards for lipidomic analysis. Finally, centrifuge 10 min at 12,000 rpm, 4 °C; transfer the supernatant for LC–MS analysis. Separation is performed by the Ultimate 3000 LC combined with Q Exactive MS (Thermo, Waltham, MA, USA) and screened with ESI-MS. The LC system is comprised of ACQUITY UPLC BEH C_18_ (100 mm × 2.1 mm × 1.7 μm) with Ultimate 3000 LC. The mobile phase is composed of solvent A (60% acetonitrile + 40% H_2_O + 10 mM Ammonium formate) and solvent B (10% acetonitrile + 90% isopropyl alcohol + 10 mM Ammonium formate) with a gradient elution (0–10.5 min, 30–100% B; 10.5–12.5 min, 100% B; 12.5–12.51 min, 100–30% B; 12.51–16.0 min, 30% B). The flow rate of the mobile phase is 0.3 mL·min^−1^. The column temperature is maintained at 40 °C, and the sample manager temperature is set at 4 °C.

Mass spectrometry parameters in electrospray ionization (ESI) negative mode are listed as follows: ESI-: Heater Temp 300 °C, Sheath Gas Flow rate, 45 arb; Aux Gas Flow Rate, 15 arb; Sweep Gas Flow Rate, 1 arb; spray voltage, 3.2 KV; Capillary Temp, 350 °C; S-Lens RF Level, 60%.

### 2.4. Study Design

**Figure 1:**
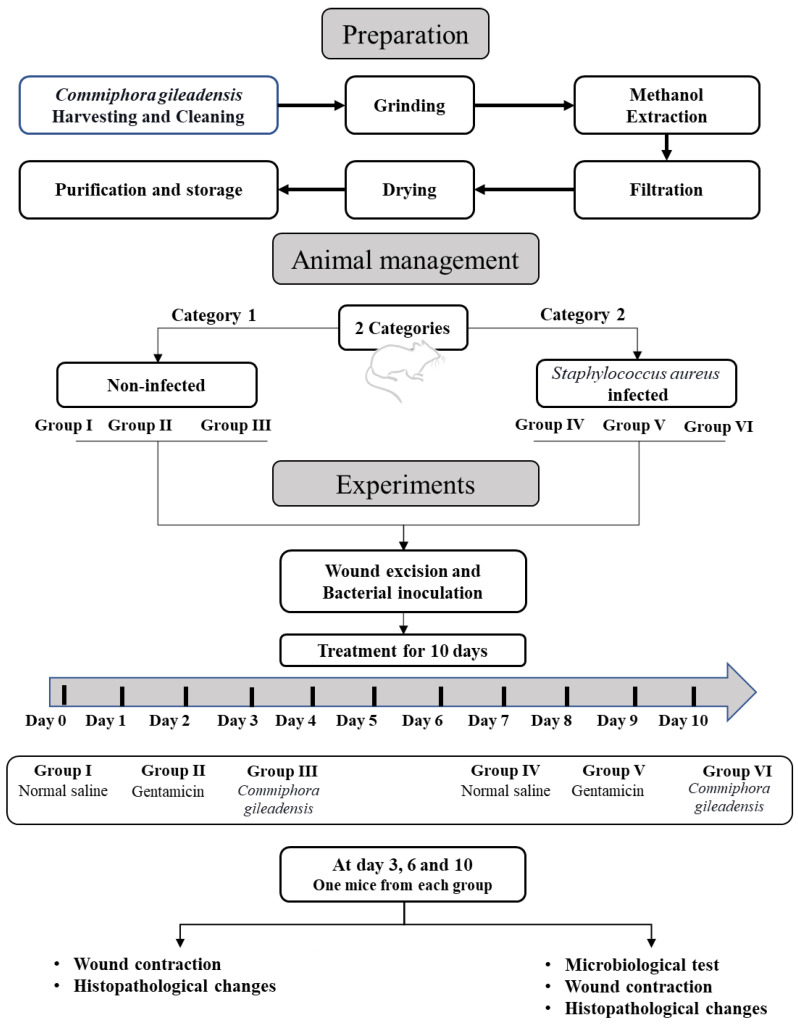
The overall work design of our pipeline.

A total of 60 10-week-old male Balb/c mice with 20–25 g body weight were obtained from Umm Al-Qura University, Saudi Arabia. Mice were kept in an ordinary rodent cage with wood chip bedding in a large, ventilated room with a 12-h light/dark cycle and a temperature of 25 ± 2 °C and received a standard rodent diet and water. After two weeks of acclimatization, mice were randomly allocated into six groups of 10 mice each. Group I was the control group, group II was assigned as a gentamicin-treated group. Group III was the *CG*-methanolic extract-treated group. Group IV was the *Staphylococcus aureus* control inoculated group, and group V was the *Staphylococcus aureus,* gentamicin-treated group. Group VI was assigned as the *Staphylococcus aureus* *CG*-methanolic extract treated group (Figure 1). The study was performed in the applied medical sciences department at Taif University and faculty of applied medical sciences, Umm Al-Qura University, Saudi Arabia. The Biomedical Research Ethics Committee approved from Umm Al-Qura University, Makkah, Saudi Arabia, with approval no. HAPO-02-K-012-2021-10-784.

### 2.5. Excision Wound Model

On the day of wound excision, mice were anesthetized by intramuscular injection of ketamine and diazepam 50 and 5 mg/kg, respectively. Hair was clipped from the distal part of the mice’s backs, where one full-thickness rounded excisional skin wound of six mm in diameter was aseptically induced in all mice under disinfected conditions [35]. The wound was kept uncovered during the experiment.

### 2.6. Bacterial Inoculation

After wound excision, an inoculum of *Staphylococcus aureus* suspension containing 10^6^ CFU/mL was immediately applied on the wound surface on each mouse in the fourth, fifth and sixth group using a sterile loop [36].

### 2.7. Treatment Applications

After six hours of *Staphylococcus aureus* inoculation, the first and fourth group wounds were topically covered with normal saline. According to the body weight, 3 mg/g of gentamicin, which dissolved in sterile distilled water, was daily topically applied to the wound of the second and fifth group mice and 4 mg/g of *CG* methanolic extract, which dissolved in sterile distilled water, was also topically applied to the wound of the third and sixth groups. The mice’s wounds of the first and fourth groups were untreated.

### 2.8. Wound Contraction Percentage

The wound areas of all mice were measured in millimetres (mm) with a calliper on the third, sixth and tenth-day post-excision. The percentage of wound contraction was calculated using the following formula [37]: Percentage of wound contraction = (1 − area on day X)/(area on day 0) × 100.

### 2.9. Histopathological Study

On the third, sixth and tenth days, one mouse from each group was randomly selected and euthanized. The wounds tissues were cut off, fixed in buffered formalin for at least 24 h, and then transferred to 70% ethanol [38,39]. Tissues were processed, embedded in paraffin blocks, sectioned at ~five μm, stained with haematoxylin and eosin [40] and examined microscopically to evaluate the histopathological changes. The grade of wound healing was semi-quantitatively assessed according to 5 parameters: (i) granulation tissue, (ii) fibroblast, (iii) polymorph leukocytes, (iv) collagen deposition and (v) re-epithelization evidence. It was evaluated as absent = 0, mild = 1, moderate = 2 and marked = 3 [41]. The wound healing process outcome was described as:

1. Complete healing: there was a complete re-epithelization, a moderate granulation tissue formation, a presence of collagen fiber, and mild infiltration of polymorph leukocytes;
2. Incomplete healing: characterized by incomplete re-epithelization, a mild formation of granulation tissue, a presence of collagen fibers, and mild infiltration of polymorph leukocytes;
3. No healing: the absence of re-epithelization, granulation tissue formation, and collagen fibers, with marked polymorph leukocyte infiltration [33,42].

### 2.10. Microbiological Test

Swab samples were obtained from the *Staphylococcus aureus*-infected wounds (groups four, five, and six) on the third, sixth, and tenth days. The swabs were cultured and incubated for 24 h. The number of bacteria per sample was then counted, colony-forming units (CFUs), as described previously [43].

### 2.11. Statistical Analysis

Statistical analysis was performed using Statistical Package for Social Sciences (SPSS) version 16 (SPSS Inc., Chicago, IL, USA). All data were presented as mean ± standard of the mean (SEM). Bootstrapping was performed for small groups to the 1000 sample size. One-way analysis of variance (ANOVA) was used to compare the wound closure and microbiological test percentage. Histopathological parameters were compared using chi-square (χ^2^) tests. The level of significance was set at *p* < 0.05.

## 3. Results

### 3.1. LC–MS of CG-Methanolic Extracts

**Figure A1:**
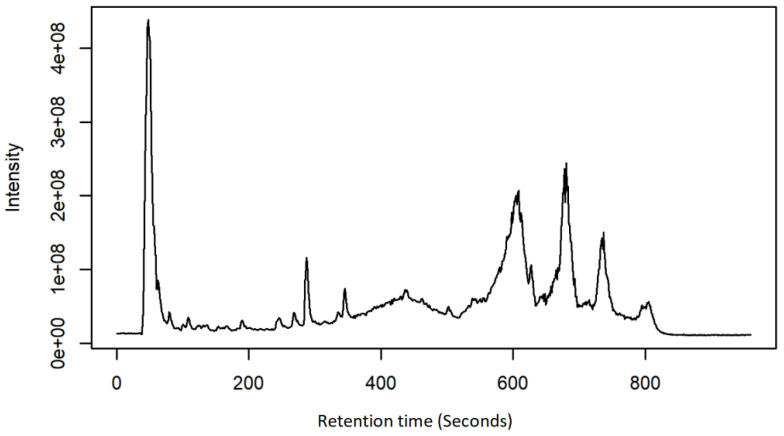
Total ion chromatogram obtained from *CG*-methanolic extracts as identified by LC–MS in negative electrospray ionization (ESI-) mode. Intensity is measured in counts per second.

**Figure A2:**
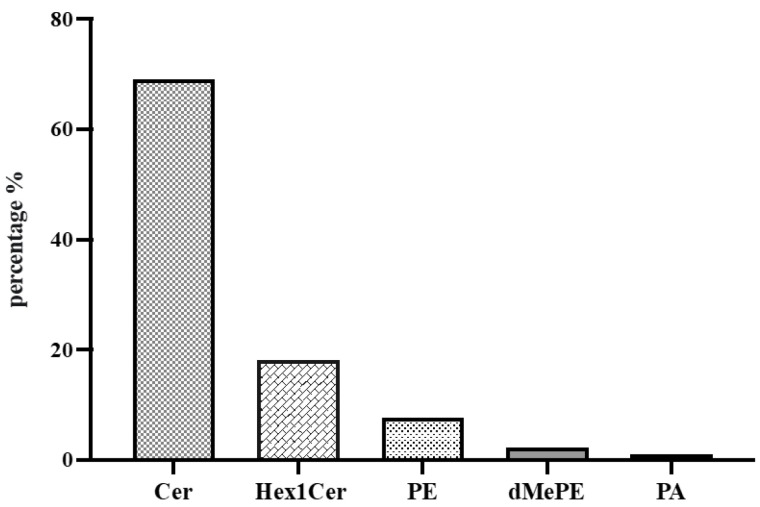
A schematic illustration of the ESI-mode profile yielded by the *CG*-methanolic extracts by LC-ESI-MS.

**Figure A3:**
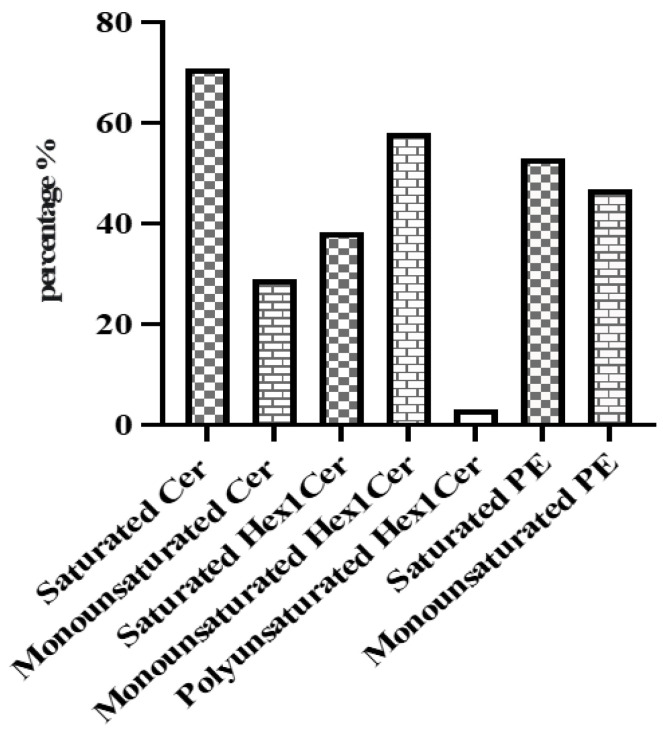
A schematic illustration of saturated and unsaturated lipid extracted of the *CG*-methanolic extracts by LC–ESI–MS.

LC–MS characterized the methanolic extracts of *CG*. The separation and detection of variable lipid species were measured and compared with internal standards using retention time (see Appendix A Figure A1) and ion formula (Table S1). The LC–MS chromatographic profile on the negative mode revealed the presence of several lipid components (Table 1). Highly abundant lipids components were detected are ceramide (Cer) 69%, hexosylceramide (Hex1Cer) 18% and phosphatidylethanolamine (PE) 7.6%, and low abundance of other components such as dimethylphosphatidylethanolamine (dMePE) 2% and phosphatidic acid (PA) 0.97% amongst others (see Appendix A Figure A2). Specifically, high monounsaturated fatty acid levels in Cer, Hex1Cer and PE, where only Hex1Cer show a small amount of polyunsaturated fatty acids (see Appendix A Figure A3).

### 3.2. Wound Healing

**Figure 2:**
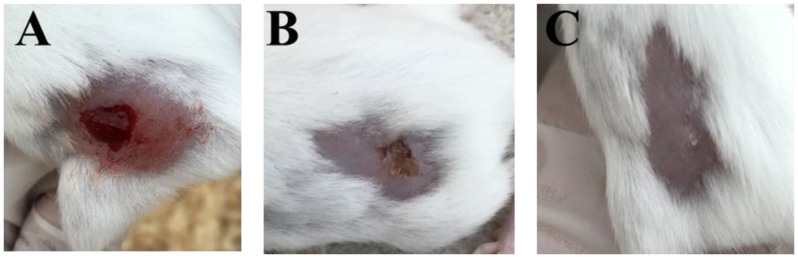
*CG*-methanolic extracts on the non-infected mice on skin excision wound through 10 days, showing better wound contraction in *CG*-methanolic extracts treated mice. (**A**) at day 0, (**B**) control at day 10 and (**C**) *CG*-methanolic extracts treated mice at day 10.

**Figure 3:**
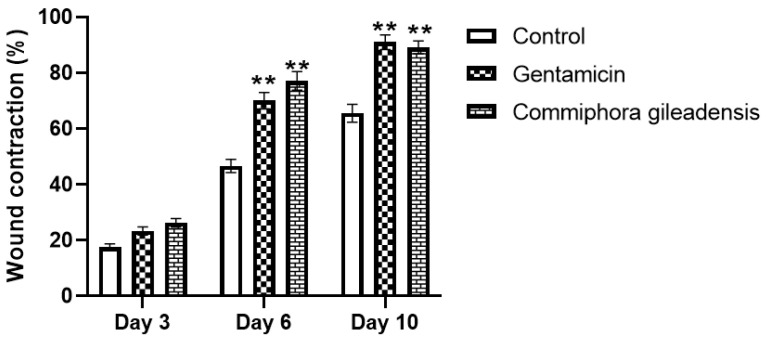
Percentage of wound contraction for control, gentamicin, and *CG*-treated mice at 3, 6 and 10 days after wounding. Results are expressed on means ± SEM of *n* = 10 mice per group. Difference is significant which ** *p*< 0.01 control vs. treated.

**Figure 4:**
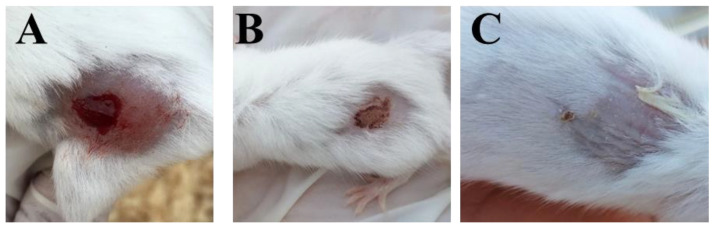
Effect of *CG*-methanolic extracts on *Staphylococcus* *aureus* infected mice. (**A**) at day 0, (**B**) control at day 6 and (**C**) *CG*-methanolic extracts treated mice at day 10.

**Figure 5:**
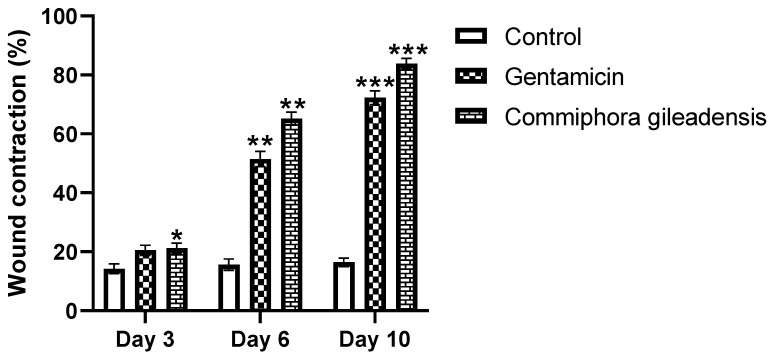
Percentage of wound contraction for control, gentamicin, and CG treated mice at 3, 6 and 10 days after wounding and Staphylococcus aureus infection. Results are expressed on means ± SEM of *n* = 10 mice per group. Difference is significant which * *p*< 0.05; ** *p*< 0.01; *** *p* < 0.001 control treated.

The percentage of wound contraction through the experiment period is summarized. The topical application of the *CG*-methanolic pure extracts at 4 mg/g promotes cutaneous wound healing by stimulating wound contraction. The percentage of wound contraction was significantly higher in the *CG*-methanolic extracts treated mice compared to the control group on the sixth and tenth days after treatment (*p* < 0.01) (Figure 2 and Figure 3). In *Staphylococcus aureus*-infected wounds, the percentage of wound contraction was significantly higher in *CG*-methanolic extract-treated mice than in the control group on the sixth and tenth days (*p* < 0.05) (Figure 4 and Figure 5).

### 3.3. Histopathological Changes

**Figure 6:**
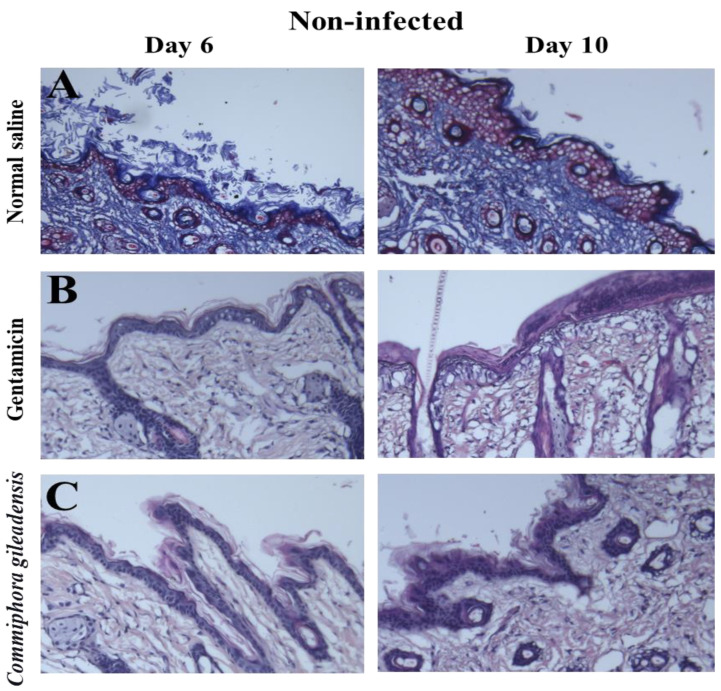
Histological changes of the 6 and 10 days, respectively, wounded skin treated topically with (**A**) normal saline, (**B**) gentamicin and (**C**) with *CG*-methanolic extracts.

Based on histopathological changes in wounds of control, gentamicin- and *CG*-methanolic extracts treated mice, on the third day, the inflammatory cell infiltration was significantly moderate in *CG*-treated mice compared to the control group (*p* < 0.01), which was milder in *CG*-methanolic extracts treated mice on the sixth and tenth days than in the control group (*p* < 0.01). Moreover, the presence of collagen fibers was significantly higher in the *CG*-methanolic extracts treated group than the control group on the sixth and tenth days (*p* < 0.01). Granulation tissue formation was also significantly higher in the *CG*-methanolic extracts treated group than in the control group on the sixth and tenth days (*p* < 0.01). On the sixth and tenth days, re-epithelization was higher in the *CG*-methanolic extracts treated mice than in the control group (*p* < 0.01). The histopathological changes post-infection with *Staphylococcus aureus* showed that the inflammatory cell infiltration on the third day was moderated in *CG*-methanolic extracts treated mice compared to the control (*p* < 0.01). It was milder on the sixth and tenth days (*p* < 0.01). Furthermore, granulation tissue formation on the sixth and tenth days was higher in *CG*-methanolic extracts treated mice compared to control (*p* < 0.01). Re-epithelisation was higher in *CG*-methanolic-extract-treated mice compared with the control group on the sixth and tenth days (*p* < 0.01) and (*p* < 0.05) (Figure 6).

### 3.4. Colony-Forming Unit (CFU) Count

**Figure 7:**
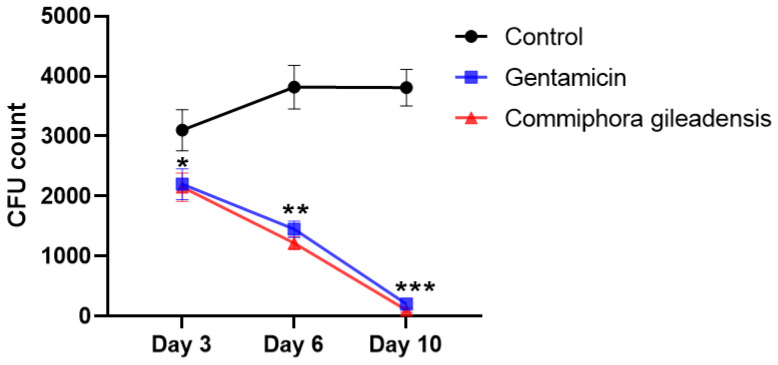
*Staphylococcus aureus* count in CFUs of control, gentamicin and *CG*-treated mice after 3, 6 and 10 days of wounding and *Staphylococcus aureus* infection. Results are expressed on means ± SEM of *n* = 10 mice per group. Difference is significant, which * *p*< 0.05; ** *p*< 0.01; *** *p* < 0.001 control vs. treated.

Figure 7 represents the *Staphylococcus aureus* count in CFU of the control, *CG*- and gentamicin-treated groups on the third, sixth and tenth days post excision and inoculation. On the third day, the CFUs of the bacteria were significantly lower in *CG*-methanolic extracts treated mice than the control group (*p* < 0.05). After the sixth and tenth days, the bacteria CFUs were significantly lowered in the *CG*-methanolic extracts treated group than in the control group (*p* < 0.01 and *p* < 0.001, respectively).

## 4. Discussion

Wound healing involves four phases haemostasis, inflammation, cell proliferation, maturation, and remodeling [8]. Historically, many plant products were used to treat different diseases and relieve many symptoms, such as Alternanthera Sessilis, morinda citrifolia, sesamum indicum, and others [44]. Saudi Arabians used *CG* for wound healing as a medicinal plant as an analgesic drug [45]. The present study used *CG*-methanolic extract to evaluate its cutaneous wound healing efficiency. Topical application of the *CG*-methanolic extract on excision wounds in mice showed statistically significant wound area contraction compared with the control group on the sixth and tenth days of the experiment.

Previously, a study done by Al-Hazmi and his colleagues (2020) showed that the methanolic extract of *Commiphora gileadensis* has an antibacterial effect on *Methicillin-resistant Staphylococcus aureus* and *Pseudomonas aeruginosa* [31]. We observed a higher rate of wound contraction in the infected and non-infected wounds when treated with *CG*-methanolic extract than in non-treated wounds. In our study, the colonization of the infected wounds that were treated with *CG*-methanolic extract was significantly lowered than the infected wounds in the control group during the experiment. The observed higher wound contraction rate in *CG*-treated mice could be due to the antibacterial effects of this extract. Regarding the structural characterization of the extracts, it shows high amount of ceramide residues, which was previously shown to have antibacterial effects on *Neisseria* [46], ceramide extracted from *Cissus incisa* leaves that showed potent antibacterial effects against *Acinetobacter baumannii* [47], and from *Euclinia longiflora* plants which show antibacterial effects on *Streptococcus pneumoniae*, *Staphylococcus aureus*, *Klepsiella pneumoniae*, *Haemophilus influenza* and *Escherichia coli* [48].

According to the histopathological findings, the non-infected wounds treated with *CG*-methanolic extract showed moderate to mild inflammatory cell infiltration compared to the control group on the tenth day of the experiment post wound excision. Thus, it may indicate that the extract has anti-inflammatory activity. The reduction in the wound inflammatory infiltration period reduced wound healing time and the susceptibility to scar formation [49]. On the sixth day, the incomplete re-epithelization of the wound was complete in the *CG*-treated group on the tenth day. It shows to be faster than what occurred in the control group, in which re-epithelization appeared incomplete on the tenth day. The difference may have been due to the extract’s antibacterial effect and rapid wound contraction rate, which reduced the distance for migrating keratinocytes in this treated group [50].

Moreover, the formation of collagen fibers began on the third day in the *CG*-treated group. This period was shorter than the control group, where such formation started on the sixth day. Collagen fiber formation may increase wounds’ tensile strength, a factor that was not measured in the study [51]. The granulation tissue formation was more apparent on the sixth day in the *CG*-treated mice than in the control group. The histopathological examination of *Staphylococcus aureus*-infected wounds showed that the inflammatory cell infiltration in *CG*-treated mice became milder on the tenth day. This period was more extended than that for non-infected wounds; however, still shorter than for the control group. Re-epithelization and granulation tissue formation appeared on the sixth day in the *CG*-treated group, shorter than the period needed in the control group. These features suggest that *CG*-supported wound healing required less time than the untreated group. A previous study showed that *CG*-methanolic extract has flavonoids, terpenoids, phenol, tannins, alkaloid, steroids, amino acids, glycosides and saponins. Terpenoids have been reported to have an antimicrobial activity that induces re-epithelization and wound contraction [52]. In addition, flavonoids and saponins have been proposed to have wound healing activity [53]. Moreover, flavonoids and glycosides possess an antioxidant activity that prevents lipid peroxidation by induction of angiogenesis. They also have anti-inflammatory and antibacterial activities that reduce cell necrosis and fibrosis. Finally, tannins were reported to be an inducer of re-epithelization. This property may induce wound healing [54].

## 5. Conclusions

In conclusion, the *CG*-methanolic extract produces an antibacterial and anti-inflammatory activity that aids in microbial elimination and encourages the wound healing process without any interruption that would worsen the condition. This study recommends the evaluation of diabetic foot ulcer healing by *CG*-methanolic extract. Future studies consider the separation, purification, and determination of the biologically active molecules from *Commiphora gileadensis* that inhibit bacterial infection. Thus, it must be conducted to identify the active compounds and reveal the specific structure to allow further studies on the active compound to be used as an antimicrobial agent, especially in infected wounds.

## Figures and Tables

**Table 1:** Lipid classes of *CG*-methanolic extracts yielded by LC–MS.

Class	%
Ceramide (Cer)	69.15
Hexosylceramide (Hex1Cer)	18.19
Phosphatidylethanolamine (PE)	7.64
Dimethylphosphatidylethanolamine (dMePE)	2.19
Phosphatidic acid (PA)	0.97
Phosphatidylinositol (PI)	0.63
Cyclic phosphatidic acid (cPA)	0.30
Lysodimethylphosphatidylethanolamine (LdMePE)	0.30
Ceramide phosphate (CerP)	0.15
Lysophosphatidic acid (LPA)	0.10
Phosphatidylglycerol (PG)	0.08
Phosphatidylmethanol (PMe)	0.04
Lysophosphatidylinositol (LPI)	0.04
Dilysocardiolipin (DLCL)	0.03
Lysophosphatidylcholine (LPC)	0.03
(O-acyl)-1-hydroxy fatty acid (OAHFA)	0.03
Phosphatidylethanol (PEt)	0.02
Sphingosine phosphate (SPHP)	0.01
Monolysocardiolipin (MLCL)	0.01
Digalactosylmonoacylglycerol(DGMG)	0.01
Fatty acid (FA)	0.01
Phosphatidylcholine (PC)	0.01
Lysophosphatidylethanol (LPEt)	0.01
Phosphatidylserine (PS)	0.01
Lysophosphatidylglycerol (LPG)	0.01
Phosphatidylinositol-P (PIP)	0.01
Lysosphingomyelin (LSM)	0.01
Monogalactosyldiacylglycerol (MGDG)	0.01
Phosphatidylinositol-P2 (PIP-2)	0.00
Total	100.00

## Data Availability

The authors will make the raw data of this article without undue reservation.

## Supplementary Materials

The following supporting information can be downloaded at: https://www.mdpi.com/article/10.3390/molecules27103320/s1, Table S1: Untargeted lipidomic analysis of the negative ion of *CG*-methanolic extracts.

## Abbreviations

*Commiphora gileadensis* (*CG*), ceramide (Cer), hexosylceramide (Hex1Cer), phosphatidylethanolamine (PE), dimethylphosphatidylethanolamine (dMePE), phosphatidic acid (PA).
