# Supplementary material for: Antibacterial Effects of Commiphora gileadensis Methanolic Extract on Wound Healing
Source: Molecules. 2022 May 21;27(10):3320. doi: 10.3390/molecules27103320 (PMC9143547; doi:10.3390/molecules27103320)
Supplement: Supplementary file 1 [file molecules-27-03320-s001.zip › molecules-1710572-supplementary.pdf]

**Table S1.** Untargeted lipidomic analysis of the negative ion of *CG*-methanolic extracts.

| # | Lipid Ion | Lipid Group | Class | Fatty Acid | FA1 | FA2 | CalcMz | ObsMz | Rt | Ion Formula |
|---|---|---|---|---|---|---|---|---|---|---|
| 1 | Cer(d16:1_26:6)+HCOO | Cer(d42:7)+HCOO | Cer | (d16:1_26:6) | (d16:1) | (26:6) | 682.541598 | 682.5416 | 11.418 | C43 H72 O5 N1 |
| 2 | Cer(d17:0_16:0)+HCOO | Cer(d33:0)+HCOO | Cer | (d17:0_16:0) | (d17:0) | (16:0) | 570.510298 | 570.50984 | 10.73 | C34 H68 O5 N1 |
| 3 | Cer(d17:0_21:0)+HCOO | Cer(d38:0)+HCOO | Cer | (d17:0_21:0) | (d17:0) | (21:0) | 640.588548 | 640.58855 | 13.002 | C39 H78 O5 N1 |
| 4 | Cer(d18:0_16:0)-H | Cer(d34:0)-H | Cer | (d18:0_16:0) | (d18:0) | (16:0) | 538.520468 | 538.52047 | 11.733 | C34 H68 O3 N1 |
| 5 | Cer(d18:0_19:0)+HCOO | Cer(d37:0)+HCOO | Cer | (d18:0_19:0) | (d18:0) | (19:0) | 626.572898 | 626.5729 | 12.477 | C38 H76 O5 N1 |
| 6 | Cer(d18:0_21:0)+HCOO | Cer(d39:0)+HCOO | Cer | (d18:0_21:0) | (d18:0) | (21:0) | 654.604198 | 654.6042 | 7.286 | C40 H80 O5 N1 |
| 7 | Cer(d18:0_22:0)+HCOO | Cer(d40:0)+HCOO | Cer | (d18:0_22:0) | (d18:0) | (22:0) | 668.619848 | 668.619824 | 10.165 | C41 H82 O5 N1 |
| 8 | Cer(d18:0_25:0)+HCOO | Cer(d43:0)+HCOO | Cer | (d18:0_25:0) | (d18:0) | (25:0) | 710.666798 | 710.666604 | 13.311 | C44 H88 O5 N1 |
| 9 | Cer(d18:1_15:0)+HCOO | Cer(d33:1)+HCOO | Cer | (d18:1_15:0) | (d18:1) | (15:0) | 568.494648 | 568.494483 | 10.259 | C34 H66 O5 N1 |
| 10 | Cer(d18:1_19:0)+HCOO | Cer(d37:1)+HCOO | Cer | (d18:1_19:0) | (d18:1) | (19:0) | 624.557248 | 624.55725 | 11.979 | C38 H74 O5 N1 |
| 11 | Cer(d18:1_20:0)+HCOO | Cer(d38:1)+HCOO | Cer | (d18:1_20:0) | (d18:1) | (20:0) | 638.572898 | 638.5729 | 12.458 | C39 H76 O5 N1 |
| 12 | Cer(d18:1_21:0)+HCOO | Cer(d39:1)+HCOO | Cer | (d18:1_21:0) | (d18:1) | (21:0) | 652.588548 | 652.588403 | 12.968 | C40 H78 O5 N1 |
| 13 | Cer(d18:1_22:0)+HCOO | Cer(d40:1)+HCOO | Cer | (d18:1_22:0) | (d18:1) | (22:0) | 666.604198 | 666.604097 | 7.26 | C41 H80 O5 N1 |
| 14 | Cer(d18:1_23:0)+HCOO | Cer(d41:1)+HCOO | Cer | (d18:1_23:0) | (d18:1) | (23:0) | 680.619848 | 680.619706 | 10.125 | C42 H82 O5 N1 |
| 15 | Cer(d18:1_24:0)+HCOO | Cer(d42:1)+HCOO | Cer | (d18:1_24:0) | (d18:1) | (24:0) | 694.635498 | 694.635158 | 11.348 | C43 H84 O5 N1 |
| 16 | Cer(d18:1_25:0)+HCOO | Cer(d43:1)+HCOO | Cer | (d18:1_25:0) | (d18:1) | (25:0) | 708.651148 | 708.650731 | 12.204 | C44 H86 O5 N1 |
| 17 | Cer(d18:2_16:0)-H | Cer(d34:2)-H | Cer | (d18:2_16:0) | (d18:2) | (16:0) | 534.489168 | 534.488446 | 10.513 | C34 H64 O3 N1 |
| 18 | Cer(d19:0_22:0)+HCOO | Cer(d41:0)+HCOO | Cer | (d19:0_22:0) | (d19:0) | (22:0) | 682.635498 | 682.6355 | 11.349 | C42 H84 O5 N1 |
| 19 | Cer(d19:0_23:0)+HCOO | Cer(d42:0)+HCOO | Cer | (d19:0_23:0) | (d19:0) | (23:0) | 696.651148 | 696.65115 | 12.276 | C43 H86 O5 N1 |
| 20 | Cer(d19:0_23:1)+HCOO | Cer(d42:1)+HCOO | Cer | (d19:0_23:1) | (d19:0) | (23:1) | 694.635498 | 694.6355 | 9.727 | C43 H84 O5 N1 |
| 21 | Cer(d19:0_25:1)+HCOO | Cer(d44:1)+HCOO | Cer | (d19:0_25:1) | (d19:0) | (25:1) | 722.666798 | 722.6668 | 12.08 | C45 H88 O5 N1 |
| 22 | Cer(d20:1_25:0)+HCOO | Cer(d45:1)+HCOO | Cer | (d20:1_25:0) | (d20:1) | (25:0) | 736.682448 | 736.68245 | 13.11 | C46 H90 O5 N1 |
| 23 | Cer(d34:1)-H | Cer(d34:1)-H | Cer | (d34:1) | (d34:1) | | 536.504818 | 536.50482 | 11.009 | C34 H66 O3 N1 |
| 24 | Cer(d34:6)+HCOO | Cer(d34:6)+HCOO | Cer | (d34:6) | (d34:6) | | 572.432048 | 572.43205 | 11.287 | C35 H58 O5 N1 |
| 25 | Cer(d41:1)-H | Cer(d41:1)-H | Cer | (d41:1) | (d41:1) | | 634.614368 | 634.61437 | 11.978 | C41 H80 O3 N1 |
| 26 | Cer(d41:1)+HCOO | Cer(d41:1)+HCOO | Cer | (d41:1) | (d41:1) | | 680.619848 | 680.61985 | 13.32 | C42 H82 O5 N1 |

| | | | | | | | | | | |
|---|---|---|---|---|---|---|---|---|---|---|
| 27 | Cer(d44:1)+HCOO | Cer(d44:1)+HCOO | Cer | (d44:1) | (d44:1) | | 722.666798 | 722.6668 | 13.308 | C45 H88 O5 N1 |
| 28 | Cer(m17:0_24:0)+HCOO | Cer(m41:0)+HCOO | Cer | (m17:0_24:0) | (m17:0) | (24:0) | 666.640583 | 666.64058 | 11.914 | C42 H84 O4 N1 |
| 29 | Cer(m17:0_25:0)+HCOO | Cer(m42:0)+HCOO | Cer | (m17:0_25:0) | (m17:0) | (25:0) | 680.656233 | 680.65623 | 12.882 | C43 H86 O4 N1 |
| 30 | Cer(m17:1_23:0)+HCOO | Cer(m40:1)+HCOO | Cer | (m17:1_23:0) | (m17:1) | (23:0) | 650.609283 | 650.60928 | 9.462 | C41 H80 O4 N1 |
| 31 | Cer(m18:0_15:0)+HCOO | Cer(m33:0)+HCOO | Cer | (m18:0_15:0) | (m18:0) | (15:0) | 554.515383 | 554.51538 | 11.467 | C34 H68 O4 N1 |
| 32 | Cer(m18:1_15:0)+HCOO | Cer(m33:1)+HCOO | Cer | (m18:1_15:0) | (m18:1) | (15:0) | 552.499733 | 552.49973 | 11.124 | C34 H66 O4 N1 |
| 33 | Cer(m19:1_25:2)+HCOO | Cer(m44:3)+HCOO | Cer | (m19:1_25:2) | (m19:1) | (25:2) | 702.640583 | 702.64095 | 13.449 | C45 H84 O4 N1 |
| 34 | Cer(m34:2+2O)-H | Cer(m34:2+2O)-H | Cer | (m34:2+2O) | (m34:2+2O) | | 550.484083 | 550.48408 | 9.802 | C34 H64 O4 N1 |
| 35 | Cer(m45:1+O)+HCOO | Cer(m45:1+O)+HCOO | Cer | (m45:1+O) | (m45:1+O) | | 736.682448 | 736.68245 | 13.11 | C46 H90 O5 N1 |
| 36 | Cer(t16:1_17:0)+HCOO | Cer(t33:1)+HCOO | Cer | (t16:1_17:0) | (t16:1) | (17:0) | 584.489563 | 584.48956 | 8.234 | C34 H66 O6 N1 |
| 37 | Cer(t16:1_18:1)-H | Cer(t34:2)-H | Cer | (t16:1_18:1) | (t16:1) | (18:1) | 550.484083 | 550.483858 | 10.664 | C34 H64 O4 N1 |
| 38 | Cer(t17:0_21:0)-H | Cer(t38:0)-H | Cer | (t17:0_21:0) | (t17:0) | (21:0) | 610.577983 | 610.57798 | 12.777 | C38 H76 O4 N1 |
| 39 | Cer(t17:0_24:1)-H | Cer(t41:1)-H | Cer | (t17:0_24:1) | (t17:0) | (24:1) | 650.609283 | 650.60928 | 11.327 | C41 H80 O4 N1 |
| 40 | Cer(t17:0_25:0+O)-H | Cer(t42:0+O)-H | Cer | (t17:0_25:0+O) | (t17:0) | (25:0+O) | 682.635498 | 682.635158 | 11.349 | C42 H84 O5 N1 |
| 41 | Cer(t17:0_25:0+O)+HCOO | Cer(t42:0+O)+HCOO | Cer | (t17:0_25:0+O) | (t17:0) | (25:0+O) | 728.640978 | 728.64098 | 11.344 | C43 H86 O7 N1 |
| 42 | Cer(t17:1_25:0+O)+HCOO | Cer(t42:1+O)+HCOO | Cer | (t17:1_25:0+O) | (t17:1) | (25:0+O) | 726.625328 | 726.62533 | 10.111 | C43 H84 O7 N1 |
| 43 | Cer(t18:0_16:0)-H | Cer(t34:0)-H | Cer | (t18:0_16:0) | (t18:0) | (16:0) | 554.515383 | 554.51538 | 10.981 | C34 H68 O4 N1 |
| 44 | Cer(t18:0_18:0)-H | Cer(t36:0)-H | Cer | (t18:0_18:0) | (t18:0) | (18:0) | 582.546683 | 582.54668 | 11.804 | C36 H72 O4 N1 |
| 45 | Cer(t18:0_18:0+O)-H | Cer(t36:0+O)-H | Cer | (t18:0_18:0+O) | (t18:0) | (18:0+O) | 598.541598 | 598.5416 | 11.56 | C36 H72 O5 N1 |
| 46 | Cer(t18:0_20:0+O)-H | Cer(t38:0+O)-H | Cer | (t18:0_20:0+O) | (t18:0) | (20:0+O) | 626.572898 | 626.5729 | 12.471 | C38 H76 O5 N1 |
| 47 | Cer(t18:0_22:0)-H | Cer(t40:0)-H | Cer | (t18:0_22:0) | (t18:0) | (22:0) | 638.609283 | 638.60928 | 9.451 | C40 H80 O4 N1 |

| | | | | | | | | | | |
|---|---|---|---|---|---|---|---|---|---|---|
| 48 | Cer(t18:0_23:0)-H | Cer(t41:0)-H | Cer | (t18:0_23:0) | (t18:0) | (23:0) | 652.624933 | 652.62493 | 10.981 | C41 H82 O4 N1 |
| 49 | Cer(t18:0_23:0)+HCOO | Cer(t41:0)+HCOO | Cer | (t18:0_23:0) | (t18:0) | (23:0) | 698.630413 | 698.63041 | 12.397 | C42 H84 O6 N1 |
| 50 | Cer(t18:0_24:0)-H | Cer(t42:0)-H | Cer | (t18:0_24:0) | (t18:0) | (24:0) | 666.640583 | 666.640451 | 11.914 | C42 H84 O4 N1 |
| 51 | Cer(t18:0_24:1)-H | Cer(t42:1)-H | Cer | (t18:0_24:1) | (t18:0) | (24:1) | 664.624933 | 664.62493 | 12.195 | C42 H82 O4 N1 |
| 52 | Cer(t18:0_25:0)-H | Cer(t43:0)-H | Cer | (t18:0_25:0) | (t18:0) | (25:0) | 680.656233 | 680.65623 | 12.882 | C43 H86 O4 N1 |
| 53 | Cer(t18:1_16:0)-H | Cer(t34:1)-H | Cer | (t18:1_16:0) | (t18:1) | (16:0) | 552.499733 | 552.49973 | 10.68 | C34 H66 O4 N1 |
| 54 | Cer(t18:1_22:0)-H | Cer(t40:1)-H | Cer | (t18:1_22:0) | (t18:1) | (22:0) | 636.593633 | 636.59363 | 13.316 | C40 H78 O4 N1 |
| 55 | Cer(t18:1_22:0)+HCOO | Cer(t40:1)+HCOO | Cer | (t18:1_22:0) | (t18:1) | (22:0) | 682.599113 | 682.59911 | 11.561 | C41 H80 O6 N1 |
| 56 | Cer(t18:1_23:0)+HCOO | Cer(t41:1)+HCOO | Cer | (t18:1_23:0) | (t18:1) | (23:0) | 696.614763 | 696.61476 | 11.739 | C42 H82 O6 N1 |
| 57 | Cer(t18:1_24:0)-H | Cer(t42:1)-H | Cer | (t18:1_24:0) | (t18:1) | (24:0) | 664.624933 | 664.62493 | 10.959 | C42 H82 O4 N1 |
| 58 | Cer(t18:1_25:0)-H | Cer(t43:1)-H | Cer | (t18:1_25:0) | (t18:1) | (25:0) | 678.640583 | 678.64058 | 11.893 | C43 H84 O4 N1 |
| 59 | Cer(t18:1_25:0+O)-H | Cer(t43:1+O)-H | Cer | (t18:1_25:0+O) | (t18:1) | (25:0+O) | 694.635498 | 694.6355 | 9.917 | C43 H84 O5 N1 |
| 60 | Cer(t20:0_20:1+O)-H | Cer(t40:1+O)-H | Cer | (t20:0_20:1+O) | (t20:0) | (20:1+O) | 652.588548 | 652.587968 | 12.968 | C40 H78 O5 N1 |
| 61 | Cer(t20:0_20:1+O)+HCOO | Cer(t40:1+O)+HCOO | Cer | (t20:0_20:1+O) | (t20:0) | (20:1+O) | 698.594028 | 698.594198 | 12.982 | C41 H80 O7 N1 |
| 62 | Cer(t34:1+O)-H | Cer(t34:1+O)-H | Cer | (t34:1+O) | (t34:1+O) | | 568.494648 | 568.494227 | 10.261 | C34 H66 O5 N1 |
| 63 | Cer(t36:1+O)-H | Cer(t36:1+O)-H | Cer | (t36:1+O) | (t36:1+O) | | 596.525948 | 596.526091 | 11.159 | C36 H70 O5 N1 |
| 64 | Cer(t39:1)+HCOO | Cer(t39:1)+HCOO | Cer | (t39:1) | (t39:1) | | 668.583463 | 668.58346 | 11.204 | C40 H78 O6 N1 |
| 65 | Cer(t39:1+O)-H | Cer(t39:1+O)-H | Cer | (t39:1+O) | (t39:1+O) | | 638.572898 | 638.5729 | 12.458 | C39 H76 O5 N1 |
| 66 | Cer(t40:0+O)-H | Cer(t40:0+O)-H | Cer | (t40:0+O) | (t40:0+O) | | 654.604198 | 654.6042 | 7.286 | C40 H80 O5 N1 |
| 67 | Cer(t41:0+O)-H | Cer(t41:0+O)-H | Cer | (t41:0+O) | (t41:0+O) | | 668.619848 | 668.619758 | 10.165 | C41 H82 O5 N1 |
| 68 | Cer(t41:1)-H | Cer(t41:1)-H | Cer | (t41:1) | (t41:1) | | 650.609283 | 650.60928 | 9.462 | C41 H80 O4 N1 |
| 69 | Cer(t41:1+O)-H | Cer(t41:1+O)-H | Cer | (t41:1+O) | (t41:1+O) | | 666.604198 | 666.6042 | 7.26 | C41 H80 O5 N1 |
| 70 | Cer(t43:0+O)-H | Cer(t43:0+O)-H | Cer | (t43:0+O) | (t43:0+O) | | 696.651148 | 696.650182 | 12.277 | C43 H86 O5 N1 |
| 71 | Cer(t43:0+O)+HCOO | Cer(t43:0+O)+HCOO | Cer | (t43:0+O) | (t43:0+O) | | 742.656628 | 742.65663 | 12.285 | C44 H88 O7 N1 |
| 72 | Cer(t43:1+O)-H | Cer(t43:1+O)-H | Cer | (t43:1+O) | (t43:1+O) | | 694.635498 | 694.6355 | 11.348 | C43 H84 O5 N1 |
| 73 | Cer(t44:0+O)-H | Cer(t44:0+O)-H | Cer | (t44:0+O) | (t44:0+O) | | 710.666798 | 710.6668 | 13.308 | C44 H88 O5 N1 |
| 74 | Cer(t44:1+O)-H | Cer(t44:1+O)-H | Cer | (t44:1+O) | (t44:1+O) | | 708.651148 | 708.650971 | 12.204 | C44 H86 O5 N1 |

| | | | | | | | | | | |
|---|---|---|---|---|---|---|---|---|---|---|
| 75 | CerP(d33:1)+HCOO | CerP(d33:1)+HCOO | CerP | (d33:1) | (d33:1) | | 648.460981 | 648.46098 | 8.853 | C34 H67 O8 N1 P1 |
| 76 | CerP(d39:1)+HCOO | CerP(d39:1)+HCOO | CerP | (d39:1) | (d39:1) | | 732.554881 | 732.55488 | 11.511 | C40 H79 O8 N1 P1 |
| 77 | CerP(d40:1)+HCOO | CerP(d40:1)+HCOO | CerP | (d40:1) | (d40:1) | | 746.570531 | 746.57053 | 11.911 | C41 H81 O8 N1 P1 |
| 78 | CerP(d41:1)+HCOO | CerP(d41:1)+HCOO | CerP | (d41:1) | (d41:1) | | 760.586181 | 760.58618 | 12.323 | C42 H83 O8 N1 P1 |
| 79 | CerP(d41:1+O)+HCOO | CerP(d41:1+O)+HCO O | CerP | (d41:1+O) | (d41:1+O) | | 776.581096 | 776.5811 | 11.683 | C42 H83 O9 N1 P1 |
| 80 | CerP(d42:1)+HCOO | CerP(d42:1)+HCOO | CerP | (d42:1) | (d42:1) | | 774.601831 | 774.60183 | 12.795 | C43 H85 O8 N1 P1 |
| 81 | CerP(d42:1+O)+HCOO | CerP(d42:1+O)+HCO O | CerP | (d42:1+O) | (d42:1+O) | | 790.596746 | 790.59675 | 12.088 | C43 H85 O9 N1 P1 |
| 82 | CerP(m33:0+O)+HCOO | CerP(m33:0+O)+HCO O | CerP | (m33:0+O) | (m33:0+O) | | 650.476631 | 650.47663 | 9.342 | C34 H69 O8 N1 P1 |
| 83 | CerP(m39:0+O)+HCOO | CerP(m39:0+O)+HCO O | CerP | (m39:0+O) | (m39:0+O) | | 734.570531 | 734.57053 | 11.89 | C40 H81 O8 N1 P1 |
| 84 | CerP(m40:0+O)+HCOO | CerP(m40:0+O)+HCO O | CerP | (m40:0+O) | (m40:0+O) | | 748.586181 | 748.58618 | 12.303 | C41 H83 O8 N1 P1 |
| 85 | CerP(m41:0)+HCOO | CerP(m41:0)+HCOO | CerP | (m41:0) | (m41:0) | | 746.606916 | 746.60692 | 13.032 | C42 H85 O7 N1 P1 |
| 86 | CerP(m41:0+O)+HCOO | CerP(m41:0+O)+HCO O | CerP | (m41:0+O) | (m41:0+O) | | 762.601831 | 762.60183 | 12.79 | C42 H85 O8 N1 P1 |
| 87 | CerP(m42:0+O)+HCOO | CerP(m42:0+O)+HCO O | CerP | (m42:0+O) | (m42:0+O) | | 776.617481 | 776.61748 | 13.304 | C43 H87 O8 N1 P1 |
| 88 | CerP(t41:0)+HCOO | CerP(t41:0)+HCOO | CerP | (t41:0) | (t41:0) | | 778.596746 | 778.59675 | 12.084 | C42 H85 O9 N1 P1 |
| 89 | CerP(t41:0+O)-H | CerP(t41:0+O)-H | CerP | (t41:0+O) | (t41:0+O) | | 748.586181 | 748.58618 | 12.303 | C41 H83 O8 N1 P1 |
| 90 | CerP(t42:0)+HCOO | CerP(t42:0)+HCOO | CerP | (t42:0) | (t42:0) | | 792.612396 | 792.6124 | 12.541 | C43 H87 O9 N1 P1 |
| 91 | CerP(t42:0+O)-H | CerP(t42:0+O)-H | CerP | (t42:0+O) | (t42:0+O) | | 762.601831 | 762.60183 | 12.792 | C42 H85 O8 N1 P1 |
| 92 | CerP(t42:1+O)-H | CerP(t42:1+O)-H | CerP | (t42:1+O) | (t42:1+O) | | 760.586181 | 760.58618 | 12.323 | C42 H83 O8 N1 P1 |
| 93 | cPA(12:0)-H | cPA(12:0)-H | cPA | (12:0) | (12:0) | | 335.162902 | 335.162981 | 1.328 | C15 H28 O6 N0 P1 |
| 94 | cPA(14:0)-H | cPA(14:0)-H | cPA | (14:0) | (14:0) | | 363.194202 | 363.1942 | 9.572 | C17 H32 O6 N0 P1 |
| 95 | cPA(16:0)-H | cPA(16:0)-H | cPA | (16:0) | (16:0) | | 391.225502 | 391.225281 | 10.451 | C19 H36 O6 N0 P1 |
| 96 | cPA(20:0)-H | cPA(20:0)-H | cPA | (20:0) | (20:0) | | 447.288102 | 447.2881 | 12.02 | C23 H44 O6 N0 P1 |
| 97 | cPA(22:0)-H | cPA(22:0)-H | cPA | (22:0) | (22:0) | | 475.319402 | 475.3194 | 12.954 | C25 H48 O6 N0 P1 |
| 98 | cPA(24:0)-H | cPA(24:0)-H | cPA | (24:0) | (24:0) | | 503.350702 | 503.3507 | 9.735 | C27 H52 O6 N0 P1 |

| | | | | | | | | | | |
|---|---|---|---|---|---|---|---|---|---|---|
| 99 | DGMG(18:3)-H | DGMG(18:3)-H | DGMG | (18:3) | (18:3) | | 675.359734 | 675.359399 | 8.848 | C33 H55 O14 |
| 100 | DLCL(29:2)-H | DLCL(29:2)-H | DLCL | (29:2) | (29:2) | | 829.427375 | 829.42737 | 10.467 | C38 H71 O15 P2 |
| 101 | DLCL(31:2)-H | DLCL(31:2)-H | DLCL | (31:2) | (31:2) | | 857.458675 | 857.45867 | 11.248 | C40 H75 O15 P2 |
| 102 | DLCL(31:4)-H | DLCL(31:4)-H | DLCL | (31:4) | (31:4) | | 853.427375 | 853.42737 | 9.816 | C40 H71 O15 P2 |
| 103 | dMePE(15:0_18:2)-H | dMePE(33:2)-H | dMePE | (15:0_18:2) | (15:0) | (18:2) | 728.523581 | 728.52358 | 10.4 | C40 H75 O8 N1 P1 |
| 104 | dMePE(16:0_14:0)-H | dMePE(30:0)-H | dMePE | (16:0_14:0) | (16:0) | (14:0) | 690.507931 | 690.50793 | 9.82 | C37 H73 O8 N1 P1 |
| 105 | dMePE(16:0_16:0)-H | dMePE(32:0)-H | dMePE | (16:0_16:0) | (16:0) | (16:0) | 718.539231 | 718.53923 | 11.41 | C39 H77 O8 N1 P1 |
| 106 | dMePE(16:0_18:1)-H | dMePE(34:1)-H | dMePE | (16:0_18:1) | (16:0) | (18:1) | 744.554881 | 744.555323 | 11.438 | C41 H79 O8 N1 P1 |
| 107 | dMePE(16:0_18:2)-H | dMePE(34:2)-H | dMePE | (16:0_18:2) | (16:0) | (18:2) | 742.539231 | 742.539108 | 10.815 | C41 H77 O8 N1 P1 |
| 108 | dMePE(16:0_18:3)-H | dMePE(34:3)-H | dMePE | (16:0_18:3) | (16:0) | (18:3) | 740.523581 | 740.52358 | 10.225 | C41 H75 O8 N1 P1 |
| 109 | dMePE(16:0_20:4)-H | dMePE(36:4)-H | dMePE | (16:0_20:4) | (16:0) | (20:4) | 766.539231 | 766.53923 | 10.679 | C43 H77 O8 N1 P1 |
| 110 | dMePE(17:0_18:2)-H | dMePE(35:2)-H | dMePE | (17:0_18:2) | (17:0) | (18:2) | 756.554881 | 756.55488 | 11.212 | C42 H79 O8 N1 P1 |
| 111 | dMePE(18:0_16:0)-H | dMePE(34:0)-H | dMePE | (18:0_16:0) | (18:0) | (16:0) | 746.570531 | 746.57053 | 12.243 | C41 H81 O8 N1 P1 |
| 112 | dMePE(18:0_18:1)-H | dMePE(36:1)-H | dMePE | (18:0_18:1) | (18:0) | (18:1) | 772.586181 | 772.58618 | 12.263 | C43 H83 O8 N1 P1 |
| 113 | dMePE(18:0_18:2)-H | dMePE(36:2)-H | dMePE | (18:0_18:2) | (18:0) | (18:2) | 770.570531 | 770.57108 | 11.595 | C43 H81 O8 N1 P1 |
| 114 | dMePE(18:0_20:4)-H | dMePE(38:4)-H | dMePE | (18:0_20:4) | (18:0) | (20:4) | 794.570531 | 794.57053 | 11.455 | C45 H81 O8 N1 P1 |
| 115 | dMePE(18:1_18:2)-H | dMePE(36:3)-H | dMePE | (18:1_18:2) | (18:1) | (18:2) | 768.554881 | 768.554468 | 10.849 | C43 H79 O8 N1 P1 |
| 116 | dMePE(18:2_18:2)-H | dMePE(36:4)-H | dMePE | (18:2_18:2) | (18:2) | (18:2) | 766.539231 | 766.538959 | 10.175 | C43 H77 O8 N1 P1 |
| 117 | dMePE(18:3_18:2)-H | dMePE(36:5)-H | dMePE | (18:3_18:2) | (18:3) | (18:2) | 764.523581 | 764.52358 | 9.528 | C43 H75 O8 N1 P1 |
| 118 | dMePE(20:0_18:2)-H | dMePE(38:2)-H | dMePE | (20:0_18:2) | (20:0) | (18:2) | 798.601831 | 798.60183 | 11.654 | C45 H85 O8 N1 P1 |
| 119 | dMePE(28:0_13:0)-H | dMePE(41:0)-H | dMePE | (28:0_13:0) | (28:0) | (13:0) | 844.680081 | 844.68008 | 9.922 | C48 H95 O8 N1 P1 |
| 120 | dMePE(30:0_14:1)-H | dMePE(44:1)-H | dMePE | (30:0_14:1) | (30:0) | (14:1) | 884.711381 | 884.718812 | 11.96 | C51 H99 O8 N1 P1 |
| 121 | dMePE(33:5)-H | dMePE(33:5)-H | dMePE | (33:5) | (33:5) | | 722.476631 | 722.47663 | 5.505 | C40 H69 O8 N1 P1 |
| 122 | dMePE(41:0)-H | dMePE(41:0)-H | dMePE | (41:0) | (41:0) | | 844.680081 | 844.680105 | 7.305 | C48 H95 O8 N1 P1 |
| 123 | dMePE(41:1)-H | dMePE(41:1)-H | dMePE | (41:1) | (41:1) | | 842.664431 | 842.66443 | 1.323 | C48 H93 O8 N1 P1 |
| 124 | FA(20:0)-H | FA(20:0)-H | FA | (20:0) | (20:0) | | 311.295554 | 311.29555 | 8.717 | O2 H39 C20 |
| 125 | FA(20:5)-H | FA(20:5)-H | FA | (20:5) | (20:5) | | 301.217304 | 301.2173 | 3.381 | O2 H29 C20 |
| 126 | Hex1Cer(d16:1_18:1)-H | Hex1Cer(d34:2)-H | Hex1Cer | (d16:1_18:1) | (d16:1) | (18:1) | 696.541993 | 696.54199 | 10.462 | C40 H74 O8 N1 |

| | | | | | | | | | | |
|---|---|---|---|---|---|---|---|---|---|---|
| 127 | Hex1Cer(d19:0_22:1)+HCOO | Hex1Cer(d41:1)+HCOO | Hex1Cer | (d19:0_22:1) | (d19:0) | (22:1) | 842.672673 | 842.67267 | 6.699 | C48 H92 O10 N1 |
| 128 | Hex1Cer(d33:0)+HCOO | Hex1Cer(d33:0)+HCOO | Hex1Cer | (d33:0) | (d33:0) | | 732.563123 | 732.56312 | 10.287 | C40 H78 O10 N1 |
| 129 | Hex1Cer(d33:1)+HCOO | Hex1Cer(d33:1)+HCOO | Hex1Cer | (d33:1) | (d33:1) | | 730.547473 | 730.547113 | 9.801 | C40 H76 O10 N1 |
| 130 | Hex1Cer(d34:2+O)+HCOO | Hex1Cer(d34:2+O)+HCOO | Hex1Cer | (d34:2+O) | (d34:2+O) | | 758.542388 | 758.54239 | 10.082 | C41 H76 O11 N1 |
| 131 | Hex1Cer(d35:1)+HCOO | Hex1Cer(d35:1)+HCOO | Hex1Cer | (d35:1) | (d35:1) | | 758.578773 | 758.57877 | 10.744 | C42 H80 O10 N1 |
| 132 | Hex1Cer(d37:1)+HCOO | Hex1Cer(d37:1)+HCOO | Hex1Cer | (d37:1) | (d37:1) | | 786.610073 | 786.610051 | 11.549 | C44 H84 O10 N1 |
| 133 | Hex1Cer(d38:1)+HCOO | Hex1Cer(d38:1)+HCOO | Hex1Cer | (d38:1) | (d38:1) | | 800.625723 | 800.625321 | 11.911 | C45 H86 O10 N1 |
| 134 | Hex1Cer(d39:0)+HCOO | Hex1Cer(d39:0)+HCOO | Hex1Cer | (d39:0) | (d39:0) | | 816.657023 | 816.65702 | 12.925 | C46 H90 O10 N1 |
| 135 | Hex1Cer(d39:1)+HCOO | Hex1Cer(d39:1)+HCOO | Hex1Cer | (d39:1) | (d39:1) | | 814.641373 | 814.640348 | 12.388 | C46 H88 O10 N1 |
| 136 | Hex1Cer(d40:1)+HCOO | Hex1Cer(d40:1)+HCOO | Hex1Cer | (d40:1) | (d40:1) | | 828.657023 | 828.656504 | 12.811 | C47 H90 O10 N1 |
| 137 | Hex1Cer(d41:0)+HCOO | Hex1Cer(d41:0)+HCOO | Hex1Cer | (d41:0) | (d41:0) | | 844.688323 | 844.68832 | 9.922 | C48 H94 O10 N1 |
| 138 | Hex1Cer(d41:1)+HCOO | Hex1Cer(d41:1)+HCOO | Hex1Cer | (d41:1) | (d41:1) | | 842.672673 | 842.671905 | 13.321 | C48 H92 O10 N1 |
| 139 | Hex1Cer(d41:2)+HCOO | Hex1Cer(d41:2)+HCOO | Hex1Cer | (d41:2) | (d41:2) | | 840.657023 | 840.65702 | 11.666 | C48 H90 O10 N1 |
| 140 | Hex1Cer(d42:1)+HCOO | Hex1Cer(d42:1)+HCOO | Hex1Cer | (d42:1) | (d42:1) | | 856.688323 | 856.68825 | 9.617 | C49 H94 O10 N1 |
| 141 | Hex1Cer(d43:1)+HCOO | Hex1Cer(d43:1)+HCOO | Hex1Cer | (d43:1) | (d43:1) | | 870.703973 | 870.70317 | 11.021 | C50 H96 O10 N1 |
| 142 | Hex1Cer(d44:1)+HCOO | Hex1Cer(d44:1)+HCOO | Hex1Cer | (d44:1) | (d44:1) | | 884.719623 | 884.71962 | 11.96 | C51 H98 O10 N1 |
| 143 | Hex1Cer(m39:1)+HCOO | Hex1Cer(m39:1)+HCOO | Hex1Cer | (m39:1) | (m39:1) | | 798.646458 | 798.64646 | 12.565 | C46 H88 O9 N1 |
| 144 | Hex1Cer(m41:2)+HCOO | Hex1Cer(m41:2)+HCOO | Hex1Cer | (m41:2) | (m41:2) | | 824.662108 | 824.661675 | 9.669 | C48 H90 O9 N1 |

| | | | | | | | | | | |
|---|---|---|---|---|---|---|---|---|---|---|
| 145 | Hex1Cer(t16:0_18:2)-H | Hex1Cer(t34:2)-H | Hex1Cer | (t16:0_18:2) | (t16:0) | (18:2) | 712.536908 | 712.536255 | 10.1 | C40 H74 O9 N1 |
| 146 | Hex1Cer(t18:0_18:2)-H | Hex1Cer(t36:2)-H | Hex1Cer | (t18:0_18:2) | (t18:0) | (18:2) | 740.568208 | 740.568142 | 11.086 | C42 H78 O9 N1 |
| 147 | Hex1Cer(t31:1)+HCOO | Hex1Cer(t31:1)+HCOO | Hex1Cer | (t31:1) | (t31:1) | | 718.511088 | 718.51109 | 9.207 | C38 H72 O11 N1 |
| 148 | Hex1Cer(t33:1)+HCOO | Hex1Cer(t33:1)+HCOO | Hex1Cer | (t33:1) | (t33:1) | | 746.542388 | 746.54239 | 7.695 | C40 H76 O11 N1 |
| 149 | Hex1Cer(t33:2)-H | Hex1Cer(t33:2)-H | Hex1Cer | (t33:2) | (t33:2) | | 698.521258 | 698.52126 | 9.7 | C39 H72 O9 N1 |
| 150 | Hex1Cer(t34:0)-H | Hex1Cer(t34:0)-H | Hex1Cer | (t34:0) | (t34:0) | | 716.568208 | 716.56831 | 11.009 | C40 H78 O9 N1 |
| 151 | Hex1Cer(t34:1)-H | Hex1Cer(t34:1)-H | Hex1Cer | (t34:1) | (t34:1) | | 714.552558 | 714.552311 | 10.391 | C40 H76 O9 N1 |
| 152 | Hex1Cer(t34:2)+HCOO | Hex1Cer(t34:2)+HCOO | Hex1Cer | (t16:0_18:2) | (t16:0) | (18:2) | 758.542388 | 758.542573 | 10.082 | C41 H76 O11 N1 |
| 153 | Hex1Cer(t39:1)+HCOO | Hex1Cer(t39:1)+HCOO | Hex1Cer | (t39:1) | (t39:1) | | 830.636288 | 830.63629 | 10.821 | C46 H88 O11 N1 |
| 154 | Hex1Cer(t40:1)+HCOO | Hex1Cer(t40:1)+HCOO | Hex1Cer | (t40:1) | (t40:1) | | 844.651938 | 844.65194 | 11.208 | C47 H90 O11 N1 |
| 155 | Hex1Cer(t40:2)-H | Hex1Cer(t40:2)-H | Hex1Cer | (t40:2) | (t40:2) | | 796.630808 | 796.63081 | 12.817 | C46 H86 O9 N1 |
| 156 | Hex1Cer(t41:1)+HCOO | Hex1Cer(t41:1)+HCOO | Hex1Cer | (t41:1) | (t41:1) | | 858.667588 | 858.667042 | 11.58 | C48 H92 O11 N1 |
| 157 | Hex1Cer(t41:2)+HCOO | Hex1Cer(t41:2)+HCOO | Hex1Cer | (t41:2) | (t41:2) | | 856.651938 | 856.65194 | 11.614 | C48 H90 O11 N1 |
| 158 | Hex1Cer(t42:1)-H | Hex1Cer(t42:1)-H | Hex1Cer | (t42:1) | (t42:1) | | 826.677758 | 826.67776 | 10.343 | C48 H92 O9 N1 |
| 159 | Hex1Cer(t42:1)+HCOO | Hex1Cer(t42:1)+HCOO | Hex1Cer | (t42:1) | (t42:1) | | 872.683238 | 872.68324 | 11.961 | C49 H94 O11 N1 |
| 160 | Hex1Cer(t42:1+O)+HCOO | Hex1Cer(t42:1+O)+HCOO | Hex1Cer | (t42:1+O) | (t42:1+O) | | 888.678153 | 888.67815 | 13.348 | C49 H94 O12 N1 |
| 161 | Hex1Cer(t42:2)-H | Hex1Cer(t42:2)-H | Hex1Cer | (t42:2) | (t42:2) | | 824.662108 | 824.661801 | 9.117 | C48 H90 O9 N1 |
| 162 | Hex1Cer(t43:1)+HCOO | Hex1Cer(t43:1)+HCOO | Hex1Cer | (t43:1) | (t43:1) | | 886.698888 | 886.69889 | 12.427 | C50 H96 O11 N1 |
| 163 | Hex1Cer(t43:2)-H | Hex1Cer(t43:2)-H | Hex1Cer | (t43:2) | (t43:2) | | 838.677758 | 838.677297 | 10.771 | C49 H92 O9 N1 |
| 164 | LdMePE(12:0)-H | LdMePE(12:0)-H | LdMePE | (12:0) | (12:0) | | 424.246966 | 424.24668 | 1.334 | C19 H39 O7 N1 P1 |
| 165 | LdMePE(16:0)-H | LdMePE(16:0)-H | LdMePE | (16:0) | (16:0) | | 480.309566 | 480.309509 | 3.347 | C23 H47 O7 N1 P1 |
| 166 | LdMePE(18:0)-H | LdMePE(18:0)-H | LdMePE | (18:0) | (18:0) | | 508.340866 | 508.340546 | 4.999 | C25 H51 O7 N1 P1 |
| 167 | LdMePE(18:1)-H | LdMePE(18:1)-H | LdMePE | (18:1) | (18:1) | | 506.325216 | 506.32522 | 3.587 | C25 H49 O7 N1 P1 |

| | | | | | | | | | | |
|---|---|---|---|---|---|---|---|---|---|---|
| 168 | LdMePE(18:2)-H | LdMePE(18:2)-H | LdMePE | (18:2) | (18:2) | | 504.309566 | 504.30957 | 2.585 | C25 H47 O7 N1 P1 |
| 169 | LdMePE(20:0)-H | LdMePE(20:0)-H | LdMePE | (20:0) | (20:0) | | 536.372166 | 536.37217 | 5.317 | C27 H55 O7 N1 P1 |
| 170 | LPA(12:0)-H | LPA(12:0)-H | LPA | (12:0) | (12:0) | | 353.173467 | 353.17347 | 1.325 | C15 H30 O7 N0 P1 |
| 171 | LPA(16:0)-H | LPA(16:0)-H | LPA | (16:0) | (16:0) | | 409.236067 | 409.235779 | 10.457 | C19 H38 O7 N0 P1 |
| 172 | LPA(17:0)-H | LPA(17:0)-H | LPA | (17:0) | (17:0) | | 423.251717 | 423.25172 | 10.85 | C20 H40 O7 N0 P1 |
| 173 | LPA(18:0)-H | LPA(18:0)-H | LPA | (18:0) | (18:0) | | 437.267367 | 437.267075 | 4.559 | C21 H42 O7 N0 P1 |
| 174 | LPA(18:1)-H | LPA(18:1)-H | LPA | (18:1) | (18:1) | | 435.251717 | 435.251536 | 3.21 | C21 H40 O7 N0 P1 |
| 175 | LPA(18:2)-H | LPA(18:2)-H | LPA | (18:2) | (18:2) | | 433.236067 | 433.235718 | 9.813 | C21 H38 O7 N0 P1 |
| 176 | LPA(20:5)-H | LPA(20:5)-H | LPA | (20:5) | (20:5) | | 455.220417 | 455.219155 | 2.278 | C23 H36 O7 N0 P1 |
| 177 | LPA(22:0)-H | LPA(22:0)-H | LPA | (22:0) | (22:0) | | 493.329967 | 493.32997 | 12.945 | C25 H50 O7 N0 P1 |
| 178 | LPA(24:0)-H | LPA(24:0)-H | LPA | (24:0) | (24:0) | | 521.361267 | 521.36127 | 8.903 | C27 H54 O7 N0 P1 |
| 179 | LPC(12:0)+HCOO | LPC(12:0)+HCOO | LPC | (12:0) | (12:0) | | 484.268096 | 484.2681 | 1.333 | C21 H43 O9 N1 P1 |
| 180 | LPE(16:0)-H | LPE(16:0)-H | LPE | (16:0) | (16:0) | | 452.278266 | 452.27827 | 3.5 | C21 H43 O7 N1 P1 |
| 181 | LPEt(16:0)-H | LPEt(16:0)-H | LPEt | (16:0) | (16:0) | | 437.267367 | 437.26737 | 3.439 | C21 H42 O7 N0 P1 |
| 182 | LPEt(18:0)-H | LPEt(18:0)-H | LPEt | (18:0) | (18:0) | | 465.298667 | 465.29867 | 1.961 | C23 H46 O7 N0 P1 |
| 183 | LPEt(18:0e)-H | LPEt(18:0e)-H | LPEt | (18:0e) | (18:0e) | | 451.319402 | 451.321686 | 4.292 | C23 H48 O6 N0 P1 |
| 184 | LPEt(18:1e)-H | LPEt(18:1e)-H | LPEt | (18:1e) | (18:1e) | | 449.303752 | 449.30375 | 1.986 | C23 H46 O6 N0 P1 |
| 185 | LPEt(22:5)-H | LPEt(22:5)-H | LPEt | (22:5) | (22:5) | | 511.283017 | 511.281693 | 2.685 | C27 H44 O7 N0 P1 |
| 186 | LPEt(28:0)-H | LPEt(28:0)-H | LPEt | (28:0) | (28:0) | | 605.455167 | 605.45517 | 8.175 | C33 H66 O7 N0 P1 |
| 187 | LPG(16:0)-H | LPG(16:0)-H | LPG | (16:0) | (16:0) | | 483.272847 | 483.27284 | 2.808 | C22 H44 O9 N0 P1 |
| 188 | LPG(18:2)-H | LPG(18:2)-H | LPG | (18:2) | (18:2) | | 507.272847 | 507.27285 | 2.154 | C24 H44 O9 N0 P1 |
| 189 | LPG(24:2)-H | LPG(24:2)-H | LPG | (24:2) | (24:2) | | 591.366747 | 591.366603 | 5.829 | C30 H56 O9 N0 P1 |
| 190 | LPI(16:0)-H | LPI(16:0)-H | LPI | (16:0) | (16:0) | | 571.288892 | 571.28889 | 2.513 | C25 H48 O12 N0 P1 |
| 191 | LPI(18:0)-H | LPI(18:0)-H | LPI | (18:0) | (18:0) | | 599.320192 | 599.32019 | 3.959 | C27 H52 O12 N0 P1 |
| 192 | LPI(18:2)-H | LPI(18:2)-H | LPI | (18:2) | (18:2) | | 595.288892 | 595.288989 | 1.933 | C27 H48 O12 N0 P1 |
| 193 | LSM(t16:1)+HCOO | LSM(t16:1)+HCOO | LSM | (t16:1) | (t16:1) | | 497.29973 | 497.300034 | 5.752 | C22 H46 O8 N2 P1 |
| 194 | MGDG(29:5)+HCOO | MGDG(29:5)+HCOO | MGDG | (29:5) | (29:5) | | 723.432504 | 723.4325 | 7.887 | C39 H63 O12 |
| 195 | MGDG(41:4)-H | MGDG(41:4)-H | MGDG | (41:4) | (41:4) | | 847.630474 | 847.63047 | 12.195 | C50 H87 O10 |
| 196 | MGDG(42:4)-H | MGDG(42:4)-H | MGDG | (42:4) | (42:4) | | 861.646124 | 861.64612 | 12.542 | C51 H89 O10 |

| | | | | | | | | | | |
|---|---|---|---|---|---|---|---|---|---|---|
| 197 | MGDG(43:8)+HCOO | MGDG(43:8)+HCOO | MGDG | (43:8) | (43:8) | | 913.604654 | 913.60465 | 10.244 | C53 H85 O12 |
| 198 | MGDG(45:5)+HCOO | MGDG(45:5)+HCOO | MGDG | (45:5) | (45:5) | | 947.682904 | 947.679646 | 4.092 | C55 H95 O12 |
| 199 | MGDG(47:12)-H | MGDG(47:12)-H | MGDG | (47:12) | (47:12) | | 915.599174 | 915.59917 | 10.815 | C56 H83 O10 |
| 200 | MGDG(47:6)+HCOO | MGDG(47:6)+HCOO | MGDG | (47:6) | (47:6) | | 973.698554 | 973.69855 | 5.808 | C57 H97 O12 |
| 201 | MGDG(8:0_10:3)-H | MGDG(18:3)-H | MGDG | (8:0_10:3) | (8:0) | (10:3) | 527.286174 | 527.28617 | 1.58 | C27 H43 O10 |
| 202 | MGMG(28:0)+HCOO | MGMG(28:0)+HCOO | MGMG | (28:0) | (28:0) | | 705.515839 | 705.512758 | 8.916 | C38 H73 O11 |
| 203 | MLCL(40:3)-H | MLCL(40:3)-H | MLCL | (40:3) | (40:3) | | 995.56314 | 995.568364 | 9.828 | C49 H89 O16 P2 |
| 204 | MLCL(40:4)-H | MLCL(40:4)-H | MLCL | (40:4) | (40:4) | | 993.54749 | 993.55127 | 9.17 | C49 H87 O16 P2 |
| 205 | MLCL(53:6)-H | MLCL(53:6)-H | MLCL | (53:6) | (53:6) | | 1171.71964 | 1171.72348 | 4.583 | C62 H109 O16 P2 |
| 206 | MLCL(54:6)-H | MLCL(54:6)-H | MLCL | (54:6) | (54:6) | | 1185.73529 | 1185.73388 | 12.113 | C63 H111 O16 P2 |
| 207 | OAHFA(18:2_22:0)-H | OAHFA(40:2)-H | OAHFA | (18:2_22:0) | (18:2) | (22:0) | 617.551434 | 617.550663 | 12.847923 3 | C40 H73 O4 |
| 208 | OAHFA(34:2)-H | OAHFA(34:2)-H | OAHFA | (34:2) | (34:2) | | 533.457534 | 533.454545 | 5.618 | C34 H61 O4 |
| 209 | OAHFA(38:2)-H | OAHFA(38:2)-H | OAHFA | (38:2) | (38:2) | | 589.520134 | 589.517617 | 7.261 | C38 H69 O4 |
| 210 | OAHFA(42:2)-H | OAHFA(42:2)-H | OAHFA | (42:2) | (42:2) | | 645.582734 | 645.580211 | 8.7120200 4 | C42 H77 O4 |
| 211 | OAHFA(42:6)-H | OAHFA(42:6)-H | OAHFA | (42:6) | (42:6) | | 637.520134 | 637.52013 | 11.495 | C42 H69 O4 |
| 212 | OAHFA(44:6)-H | OAHFA(44:6)-H | OAHFA | (44:6) | (44:6) | | 665.551434 | 665.55143 | 12.396 | C44 H73 O4 |
| 213 | OAHFA(46:2)-H | OAHFA(46:2)-H | OAHFA | (46:2) | (46:2) | | 701.645334 | 701.6427 | 9.952 | C46 H85 O4 |
| 214 | OAHFA(48:6)-H | OAHFA(48:6)-H | OAHFA | (48:6) | (48:6) | | 721.614034 | 721.616358 | 6.959 | C48 H81 O4 |
| 215 | OAHFA(52:6)-H | OAHFA(52:6)-H | OAHFA | (52:6) | (52:6) | | 777.676634 | 777.677572 | 8.353 | C52 H89 O4 |
| 216 | OAHFA(56:6)-H | OAHFA(56:6)-H | OAHFA | (56:6) | (56:6) | | 833.739234 | 833.74086 | 9.5329544 6 | C56 H97 O4 |
| 217 | PA(10:0e_10:0)-H | PA(20:0e)-H | PA | (10:0e_10:0) | (10:0e) | (10:0) | 465.298667 | 465.29867 | 12.003 | C23 H46 O7 N0 P1 |
| 218 | PA(12:0_20:4)-H | PA(32:4)-H | PA | (12:0_20:4) | (12:0) | (20:4) | 639.403132 | 639.402617 | 8.253 | C35 H60 O8 N0 P1 |
| 219 | PA(14:0_18:2)-H | PA(32:2)-H | PA | (14:0_18:2) | (14:0) | (18:2) | 643.434432 | 643.43443 | 9.548 | C35 H64 O8 N0 P1 |
| 220 | PA(15:0_16:0)-H | PA(31:0)-H | PA | (15:0_16:0) | (15:0) | (16:0) | 633.450082 | 633.45008 | 10.635 | C34 H66 O8 N0 P1 |
| 221 | PA(15:0_18:2)-H | PA(33:2)-H | PA | (15:0_18:2) | (15:0) | (18:2) | 657.450082 | 657.449887 | 10.024 | C36 H66 O8 N0 P1 |
| 222 | PA(15:0_18:3)-H | PA(33:3)-H | PA | (15:0_18:3) | (15:0) | (18:3) | 655.434432 | 655.43443 | 9.368 | C36 H64 O8 N0 P1 |
| 223 | PA(16:0_14:0)-H | PA(30:0)-H | PA | (16:0_14:0) | (16:0) | (14:0) | 619.434432 | 619.43443 | 10.225 | C33 H64 O8 N0 P1 |

| | | | | | | | | | | |
|---|---|---|---|---|---|---|---|---|---|---|
| 224 | PA(16:0_16:0)-H | PA(32:0)-H | PA | (16:0_16:0) | (16:0) | (16:0) | 647.465732 | 647.464822 | 11.052 | C35 H68 O8 N0 P1 |
| 225 | PA(16:0_16:1)-H | PA(32:1)-H | PA | (16:0_16:1) | (16:0) | (16:1) | 645.450082 | 645.45008 | 10.339 | C35 H66 O8 N0 P1 |
| 226 | PA(16:0_17:0)-H | PA(33:0)-H | PA | (16:0_17:0) | (16:0) | (17:0) | 661.481382 | 661.48138 | 11.425 | C36 H70 O8 N0 P1 |
| 227 | PA(16:0_18:1)-H | PA(34:1)-H | PA | (16:0_18:1) | (16:0) | (18:1) | 673.481382 | 673.480836 | 11.089 | C37 H70 O8 N0 P1 |
| 228 | PA(16:0_18:2)-H | PA(34:2)-H | PA | (16:0_18:2) | (16:0) | (18:2) | 671.465732 | 671.465355 | 10.463 | C37 H68 O8 N0 P1 |
| 229 | PA(16:0_18:3)-H | PA(34:3)-H | PA | (16:0_18:3) | (16:0) | (18:3) | 669.450082 | 669.449678 | 9.844 | C37 H66 O8 N0 P1 |
| 230 | PA(16:0_20:5)-H | PA(36:5)-H | PA | (16:0_20:5) | (16:0) | (20:5) | 693.450082 | 693.45008 | 10.426 | C39 H66 O8 N0 P1 |
| 231 | PA(17:0_18:1)-H | PA(35:1)-H | PA | (17:0_18:1) | (17:0) | (18:1) | 687.497032 | 687.49703 | 11.456 | C38 H72 O8 N0 P1 |
| 232 | PA(17:0_18:2)-H | PA(35:2)-H | PA | (17:0_18:2) | (17:0) | (18:2) | 685.481382 | 685.481168 | 10.864 | C38 H70 O8 N0 P1 |
| 233 | PA(17:0_18:3)-H | PA(35:3)-H | PA | (17:0_18:3) | (17:0) | (18:3) | 683.465732 | 683.46573 | 10.308 | C38 H68 O8 N0 P1 |
| 234 | PA(17:1_16:0)-H | PA(33:1)-H | PA | (17:1_16:0) | (17:1) | (16:0) | 659.465732 | 659.46573 | 10.699 | C36 H68 O8 N0 P1 |
| 235 | PA(18:0_16:0)-H | PA(34:0)-H | PA | (18:0_16:0) | (18:0) | (16:0) | 675.497032 | 675.49703 | 11.811 | C37 H72 O8 N0 P1 |
| 236 | PA(18:0_18:1)-H | PA(36:1)-H | PA | (18:0_18:1) | (18:0) | (18:1) | 701.512682 | 701.51268 | 11.848 | C39 H74 O8 N0 P1 |
| 237 | PA(18:0_18:2)-H | PA(36:2)-H | PA | (18:0_18:2) | (18:0) | (18:2) | 699.497032 | 699.496763 | 11.251 | C39 H72 O8 N0 P1 |
| 238 | PA(18:0_18:3)-H | PA(36:3)-H | PA | (18:0_18:3) | (18:0) | (18:3) | 697.481382 | 697.48138 | 10.503 | C39 H70 O8 N0 P1 |
| 239 | PA(18:1_22:0)-H | PA(40:1)-H | PA | (18:1_22:0) | (18:1) | (22:0) | 757.575282 | 757.57528 | 9.431 | C43 H82 O8 N0 P1 |
| 240 | PA(18:2_18:2)-H | PA(36:4)-H | PA | (18:2_18:2) | (18:2) | (18:2) | 695.465732 | 695.465567 | 9.816 | C39 H68 O8 N0 P1 |
| 241 | PA(18:2_21:0)-H | PA(39:2)-H | PA | (18:2_21:0) | (18:2) | (21:0) | 741.543982 | 741.543897 | 12.469 | C42 H78 O8 N0 P1 |
| 242 | PA(18:2_23:0)-H | PA(41:2)-H | PA | (18:2_23:0) | (18:2) | (23:0) | 769.575282 | 769.57528 | 7.359 | C44 H82 O8 N0 P1 |
| 243 | PA(18:3_18:2)-H | PA(36:5)-H | PA | (18:3_18:2) | (18:3) | (18:2) | 693.450082 | 693.449986 | 9.156 | C39 H66 O8 N0 P1 |
| 244 | PA(18:3_18:3)-H | PA(36:6)-H | PA | (18:3_18:3) | (18:3) | (18:3) | 691.434432 | 691.43411 | 8.44 | C39 H64 O8 N0 P1 |
| 245 | PA(18:4e)-H | PA(18:4e)-H | PA | (18:4e) | (18:4e) | | 429.204767 | 429.20477 | 6.322 | C21 H34 O7 N0 P1 |
| 246 | PA(19:0_18:2)-H | PA(37:2)-H | PA | (19:0_18:2) | (19:0) | (18:2) | 713.512682 | 713.51268 | 11.631 | C40 H74 O8 N0 P1 |
| 247 | PA(20:0_16:0)-H | PA(36:0)-H | PA | (20:0_16:0) | (20:0) | (16:0) | 703.528332 | 703.52833 | 12.751 | C39 H76 O8 N0 P1 |
| 248 | PA(20:0_18:1)-H | PA(38:1)-H | PA | (20:0_18:1) | (20:0) | (18:1) | 729.543982 | 729.54398 | 12.757 | C41 H78 O8 N0 P1 |
| 249 | PA(20:0_18:2)-H | PA(38:2)-H | PA | (20:0_18:2) | (20:0) | (18:2) | 727.528332 | 727.52833 | 12.003 | C41 H76 O8 N0 P1 |
| 250 | PA(20:0_18:3)-H | PA(38:3)-H | PA | (20:0_18:3) | (20:0) | (18:3) | 725.512682 | 725.51268 | 11.259 | C41 H74 O8 N0 P1 |
| 251 | PA(22:0_18:2)-H | PA(40:2)-H | PA | (22:0_18:2) | (22:0) | (18:2) | 755.559632 | 755.55963 | 12.945 | C43 H80 O8 N0 P1 |
| 252 | PA(22:0_18:3)-H | PA(40:3)-H | PA | (22:0_18:3) | (22:0) | (18:3) | 753.543982 | 753.54398 | 12.253 | C43 H78 O8 N0 P1 |

| | | | | | | | | | | |
|---|---|---|---|---|---|---|---|---|---|---|
| 253 | PA(24:0_18:2)-H | PA(42:2)-H | PA | (24:0_18:2) | (24:0) | (18:2) | 783.590932 | 783.59093 | 10.12 | C45 H84 O8 N0 P1 |
| 254 | PA(24:0_18:3)-H | PA(42:3)-H | PA | (24:0_18:3) | (24:0) | (18:3) | 781.575282 | 781.575146 | 13.265 | C45 H82 O8 N0 P1 |
| 255 | PA(24:0_22:6)-H | PA(46:6)-H | PA | (24:0_22:6) | (24:0) | (22:6) | 831.590932 | 831.594135 | 8.406 | C49 H84 O8 N0 P1 |
| 256 | PA(25:0_18:2)-H | PA(43:2)-H | PA | (25:0_18:2) | (25:0) | (18:2) | 797.606582 | 797.60658 | 11.024 | C46 H86 O8 N0 P1 |
| 257 | PA(4:0_14:1)-H | PA(18:1)-H | PA | (4:0_14:1) | (4:0) | (14:1) | 449.230982 | 449.23098 | 1.037 | C21 H38 O8 N0 P1 |
| 258 | PA(4:0_14:2)-H | PA(18:2)-H | PA | (4:0_14:2) | (4:0) | (14:2) | 447.215332 | 447.21533 | 6.194 | C21 H36 O8 N0 P1 |
| 259 | PA(8:0_10:1)-H | PA(18:1)-H | PA | (8:0_10:1) | (8:0) | (10:1) | 449.230982 | 449.230923 | 7.652 | C21 H38 O8 N0 P1 |
| 260 | PA(8:0e_10:0)-H | PA(18:0e)-H | PA | (8:0e_10:0) | (8:0e) | (10:0) | 437.267367 | 437.26737 | 4.559 | C21 H42 O7 N0 P1 |
| 261 | PA(8:0e_10:3)-H | PA(18:3e)-H | PA | (8:0e_10:3) | (8:0e) | (10:3) | 431.220417 | 431.22042 | 1.701 | C21 H36 O7 N0 P1 |
| 262 | PA(8:0e_10:4)-H | PA(18:4e)-H | PA | (8:0e_10:4) | (8:0e) | (10:4) | 429.204767 | 429.20477 | 7.047 | C21 H34 O7 N0 P1 |
| 263 | PA(8:0e_8:0)-H | PA(16:0e)-H | PA | (8:0e_8:0) | (8:0e) | (8:0) | 409.236067 | 409.23607 | 11.827 | C19 H38 O7 N0 P1 |
| 264 | PA(8:1e_10:0)-H | PA(18:1e)-H | PA | (8:1e_10:0) | (8:1e) | (10:0) | 435.251717 | 435.25172 | 11.082 | C21 H40 O7 N0 P1 |
| 265 | PA(8:1e_10:1)-H | PA(18:2e)-H | PA | (8:1e_10:1) | (8:1e) | (10:1) | 433.236067 | 433.23607 | 7.256 | C21 H38 O7 N0 P1 |
| 266 | PA(8:1e_10:3)-H | PA(18:4e)-H | PA | (8:1e_10:3) | (8:1e) | (10:3) | 429.204767 | 429.20477 | 7.723 | C21 H34 O7 N0 P1 |
| 267 | PC(16:0_18:2)+HCOO | PC(34:2)+HCOO | PC | (16:0_18:2) | (16:0) | (18:2) | 802.560361 | 802.56036 | 10.821 | C43 H81 O10 N1 P1 |
| 268 | PC(18:0_18:2)+HCOO | PC(36:2)+HCOO | PC | (18:0_18:2) | (18:0) | (18:2) | 830.591661 | 830.59166 | 11.598 | C45 H85 O10 N1 P1 |
| 269 | PC(18:2_18:2)+HCOO | PC(36:4)+HCOO | PC | (18:2_18:2) | (18:2) | (18:2) | 826.560361 | 826.56036 | 10.174 | C45 H81 O10 N1 P1 |
| 270 | PE(16:0_16:0)-H | PE(32:0)-H | PE | (16:0_16:0) | (16:0) | (16:0) | 690.507931 | 690.50793 | 10.013 | C37 H73 O8 N1 P1 |
| 271 | PE(16:0_18:2)-H | PE(34:2)-H | PE | (16:0_18:2) | (16:0) | (18:2) | 714.507931 | 714.50807 | 9.436 | C39 H73 O8 N1 P1 |
| 272 | PE(17:0_18:2)-H | PE(35:2)-H | PE | (17:0_18:2) | (17:0) | (18:2) | 728.523581 | 728.52358 | 9.744 | C40 H75 O8 N1 P1 |
| 273 | PE(18:0_18:2)-H | PE(36:2)-H | PE | (18:0_18:2) | (18:0) | (18:2) | 742.539231 | 742.53923 | 11.818 | C41 H77 O8 N1 P1 |
| 274 | PE(18:1_18:2)-H | PE(36:3)-H | PE | (18:1_18:2) | (18:1) | (18:2) | 740.523581 | 740.52358 | 9.507 | C41 H75 O8 N1 P1 |
| 275 | PE(18:3_18:2)-H | PE(36:5)-H | PE | (18:3_18:2) | (18:3) | (18:2) | 736.492281 | 736.49228 | 8.089 | C41 H71 O8 N1 P1 |
| 276 | PE(28:0_16:0)-H | PE(44:0)-H | PE | (28:0_16:0) | (28:0) | (16:0) | 858.695731 | 858.69573 | 11.219 | C49 H97 O8 N1 P1 |
| 277 | PE(32:0_10:0)-H | PE(42:0)-H | PE | (32:0_10:0) | (32:0) | (10:0) | 830.664431 | 830.668578 | 6.713 | C47 H93 O8 N1 P1 |
| 278 | PE(32:0_11:1)-H | PE(43:1)-H | PE | (32:0_11:1) | (32:0) | (11:1) | 842.664431 | 842.66443 | 6.699 | C48 H93 O8 N1 P1 |
| 279 | PE(32:0_14:1)-H | PE(46:1)-H | PE | (32:0_14:1) | (32:0) | (14:1) | 884.711381 | 884.71138 | 11.96 | C51 H99 O8 N1 P1 |
| 280 | PE(43:0)-H | PE(43:0)-H | PE | (43:0) | (43:0) | | 844.680081 | 844.679538 | 7.305 | C48 H95 O8 N1 P1 |
| 281 | PEt(18:0e_10:1)-H | PEt(28:1e)-H | PEt | (18:0e_10:1) | (18:0e) | (10:1) | 603.439517 | 603.43952 | 7.22 | C33 H64 O7 N0 P1 |

| | | | | | | | | | | |
|---|---|---|---|---|---|---|---|---|---|---|
| 282 | PEt(35:1)-H | PEt(35:1)-H | PEt | (35:1) | (35:1) | | 715.528332 | 715.53066 | 4.315 | C40 H76 O8 N0 P1 |
| 283 | PEt(40:5)-H | PEt(40:5)-H | PEt | (40:5) | (40:5) | | 777.543982 | 777.543506 | 3.431 | C45 H78 O8 N0 P1 |
| 284 | PEt(44:4)-H | PEt(44:4)-H | PEt | (44:4) | (44:4) | | 835.622232 | 835.62223 | 12.854 | C49 H88 O8 N0 P1 |
| 285 | PEt(46:6)-H | PEt(46:6)-H | PEt | (46:6) | (46:6) | | 859.622232 | 859.626408 | 9.165 | C51 H88 O8 N0 P1 |
| 286 | PG(15:0_16:0)-H | PG(31:0)-H | PG | (15:0_16:0) | (15:0) | (16:0) | 707.486862 | 707.48686 | 10.368 | C37 H72 O10 N0 P1 |
| 287 | PG(16:0_16:0)-H | PG(32:0)-H | PG | (16:0_16:0) | (16:0) | (16:0) | 721.502512 | 721.502292 | 10.765 | C38 H74 O10 N0 P1 |
| 288 | PG(16:0_17:0)-H | PG(33:0)-H | PG | (16:0_17:0) | (16:0) | (17:0) | 735.518162 | 735.51816 | 11.129 | C39 H76 O10 N0 P1 |
| 289 | PG(16:0_18:1)-H | PG(34:1)-H | PG | (16:0_18:1) | (16:0) | (18:1) | 747.518162 | 747.518177 | 10.794 | C40 H76 O10 N0 P1 |
| 290 | PG(16:0_18:2)-H | PG(34:2)-H | PG | (16:0_18:2) | (16:0) | (18:2) | 745.502512 | 745.50251 | 10.179 | C40 H74 O10 N0 P1 |
| 291 | PG(16:0_18:3)-H | PG(34:3)-H | PG | (16:0_18:3) | (16:0) | (18:3) | 743.486862 | 743.48686 | 9.57 | C40 H72 O10 N0 P1 |
| 292 | PG(18:0_16:0)-H | PG(34:0)-H | PG | (18:0_16:0) | (18:0) | (16:0) | 749.533812 | 749.53387 | 11.496 | C40 H78 O10 N0 P1 |
| 293 | PG(18:0e_19:1)-H | PG(37:1e)-H | PG | (18:0e_19:1) | (18:0e) | (19:1) | 775.585847 | 775.585416 | 5.571 | C43 H84 O9 N0 P1 |
| 294 | PG(18:2_18:2)-H | PG(36:4)-H | PG | (18:2_18:2) | (18:2) | (18:2) | 769.502512 | 769.50251 | 9.543 | C42 H74 O10 N0 P1 |
| 295 | PG(18:3_18:2)-H | PG(36:5)-H | PG | (18:3_18:2) | (18:3) | (18:2) | 767.486862 | 767.486201 | 8.875 | C42 H72 O10 N0 P1 |
| 296 | PG(19:1_14:3)-H | PG(33:4)-H | PG | (19:1_14:3) | (19:1) | (14:3) | 727.455562 | 727.455667 | 3.638 | C39 H68 O10 N0 P1 |
| 297 | PG(20:0_11:2)-H | PG(31:2)-H | PG | (20:0_11:2) | (20:0) | (11:2) | 703.455562 | 703.45556 | 6.613 | C37 H68 O10 N0 P1 |
| 298 | PG(20:0_15:0)-H | PG(35:0)-H | PG | (20:0_15:0) | (20:0) | (15:0) | 763.549462 | 763.549 | 2.275 | C41 H80 O10 N0 P1 |
| 299 | PG(20:1_11:2)-H | PG(31:3)-H | PG | (20:1_11:2) | (20:1) | (11:2) | 701.439912 | 701.43991 | 4.582 | C37 H66 O10 N0 P1 |
| 300 | PG(24:0)-H | PG(24:0)-H | PG | (24:0) | (24:0) | | 609.377312 | 609.37731 | 5.517 | C30 H58 O10 N0 P1 |
| 301 | PG(26:3)-H | PG(26:3)-H | PG | (26:3) | (26:3) | | 631.361662 | 631.36166 | 1.531 | C32 H56 O10 N0 P1 |
| 302 | PG(27:0e)-H | PG(27:0e)-H | PG | (27:0e) | (27:0e) | | 637.444997 | 637.447244 | 6.743 | C33 H66 O9 N0 P1 |
| 303 | PG(31:3)-H | PG(31:3)-H | PG | (31:3) | (31:3) | | 701.439912 | 701.43991 | 5.474 | C37 H66 O10 N0 P1 |
| 304 | PG(32:1)-H | PG(32:1)-H | PG | (32:1) | (32:1) | | 719.486862 | 719.48686 | 11.038 | C38 H72 O10 N0 P1 |
| 305 | PG(32:3)-H | PG(32:3)-H | PG | (32:3) | (32:3) | | 715.455562 | 715.45556 | 11.059 | C38 H68 O10 N0 P1 |
| 306 | PG(33:2)-H | PG(33:2)-H | PG | (33:2) | (33:2) | | 731.486862 | 731.48686 | 7.409 | C39 H72 O10 N0 P1 |
| 307 | PG(33:3)-H | PG(33:3)-H | PG | (33:3) | (33:3) | | 729.471212 | 729.47121 | 4.695 | C39 H70 O10 N0 P1 |
| 308 | PG(33:4)-H | PG(33:4)-H | PG | (33:4) | (33:4) | | 727.455562 | 727.45556 | 5.885 | C39 H68 O10 N0 P1 |
| 309 | PG(33:5)-H | PG(33:5)-H | PG | (33:5) | (33:5) | | 725.439912 | 725.43991 | 4.327 | C39 H66 O10 N0 P1 |
| 310 | PG(34:0)-H | PG(34:0)-H | PG | (34:0) | (34:0) | | 749.533812 | 749.53381 | 6.508 | C40 H78 O10 N0 P1 |

| | | | | | | | | | | |
|---|---|---|---|---|---|---|---|---|---|---|
| 311 | PG(34:3)-H | PG(34:3)-H | PG | (34:3) | (34:3) | | 743.486862 | 743.48686 | 10.505 | C40 H72 O10 N0 P1 |
| 312 | PG(34:4)-H | PG(34:4)-H | PG | (34:4) | (34:4) | | 741.471212 | 741.47121 | 11.096 | C40 H70 O10 N0 P1 |
| 313 | PG(36:4)-H | PG(36:4)-H | PG | (36:4) | (36:4) | | 769.502512 | 769.50251 | 11.842 | C42 H74 O10 N0 P1 |
| 314 | PG(36:5)-H | PG(36:5)-H | PG | (36:5) | (36:5) | | 767.486862 | 767.48686 | 11.258 | C42 H72 O10 N0 P1 |
| 315 | PG(41:3)-H | PG(41:3)-H | PG | (41:3) | (41:3) | | 841.596412 | 841.595628 | 10.677 | C47 H86 O10 N0 P1 |
| 316 | PG(46:2)-H | PG(46:2)-H | PG | (46:2) | (46:2) | | 913.690312 | 913.69031 | 4.47 | C52 H98 O10 N0 P1 |
| 317 | PG(54:4)-H | PG(54:4)-H | PG | (54:4) | (54:4) | | 1021.78421 | 1021.78421 | 11.623 | C60 H110 O10 N0 P1 |
| 318 | PG(8:0_10:3)-H | PG(18:3)-H | PG | (8:0_10:3) | (8:0) | (10:3) | 519.236462 | 519.239685 | 13.335723<br>9 | C24 H40 O10 N0 P1 |
| 319 | PI(11:0_10:3)-H | PI(21:3)-H | PI | (11:0_10:3) | (11:0) | (10:3) | 649.299457 | 649.29946 | 1.808 | C30 H50 O13 N0 P1 |
| 320 | PI(12:0e_17:0)-H | PI(29:0e)-H | PI | (12:0e_17:0) | (12:0e) | (17:0) | 753.492342 | 753.49234 | 1.778 | C38 H74 O12 N0 P1 |
| 321 | PI(14:0e_22:2)-H | PI(36:2e)-H | PI | (14:0e_22:2) | (14:0e) | (22:2) | 847.570592 | 847.57059 | 1.783 | C45 H84 O12 N0 P1 |
| 322 | PI(15:0_18:2)-H | PI(33:2)-H | PI | (15:0_18:2) | (15:0) | (18:2) | 819.502907 | 819.50304 | 9.577 | C42 H76 O13 N0 P1 |
| 323 | PI(16:0_16:0)-H | PI(32:0)-H | PI | (16:0_16:0) | (16:0) | (16:0) | 809.518557 | 809.518566 | 10.608 | C41 H78 O13 N0 P1 |
| 324 | PI(16:0_18:1)-H | PI(34:1)-H | PI | (16:0_18:1) | (16:0) | (18:1) | 835.534207 | 835.53421 | 10.664 | C43 H80 O13 N0 P1 |
| 325 | PI(16:0_18:2)-H | PI(34:2)-H | PI | (16:0_18:2) | (16:0) | (18:2) | 833.518557 | 833.517891 | 10.01 | C43 H78 O13 N0 P1 |
| 326 | PI(16:0_18:3)-H | PI(34:3)-H | PI | (16:0_18:3) | (16:0) | (18:3) | 831.502907 | 831.503127 | 9.385 | C43 H76 O13 N0 P1 |
| 327 | PI(16:0_22:1)-H | PI(38:1)-H | PI | (16:0_22:1) | (16:0) | (22:1) | 891.596807 | 891.600717 | 11.4 | C47 H88 O13 N0 P1 |
| 328 | PI(17:0_18:2)-H | PI(35:2)-H | PI | (17:0_18:2) | (17:0) | (18:2) | 847.534207 | 847.535252 | 10.427 | C44 H80 O13 N0 P1 |
| 329 | PI(17:0_18:3)-H | PI(35:3)-H | PI | (17:0_18:3) | (17:0) | (18:3) | 845.518557 | 845.51856 | 9.848 | C44 H78 O13 N0 P1 |
| 330 | PI(18:0_16:0)-H | PI(34:0)-H | PI | (18:0_16:0) | (18:0) | (16:0) | 837.549857 | 837.54986 | 11.376 | C43 H82 O13 N0 P1 |
| 331 | PI(18:0_18:1)-H | PI(36:1)-H | PI | (18:0_18:1) | (18:0) | (18:1) | 863.565507 | 863.56551 | 11.419 | C45 H84 O13 N0 P1 |
| 332 | PI(18:0_18:2)-H | PI(36:2)-H | PI | (18:0_18:2) | (18:0) | (18:2) | 861.549857 | 861.549371 | 10.837 | C45 H82 O13 N0 P1 |
| 333 | PI(18:0_18:3)-H | PI(36:3)-H | PI | (18:0_18:3) | (18:0) | (18:3) | 859.534207 | 859.53386 | 10.055 | C45 H80 O13 N0 P1 |
| 334 | PI(18:0_20:4)-H | PI(38:4)-H | PI | (18:0_20:4) | (18:0) | (20:4) | 885.549857 | 885.550088 | 10.701 | C47 H82 O13 N0 P1 |
| 335 | PI(18:1_10:3)-H | PI(28:4)-H | PI | (18:1_10:3) | (18:1) | (10:3) | 745.393357 | 745.39336 | 6.645 | C37 H62 O13 N0 P1 |
| 336 | PI(18:1e_18:2)-H | PI(36:3e)-H | PI | (18:1e_18:2) | (18:1e) | (18:2) | 845.554942 | 845.55494 | 10.857 | C45 H82 O12 N0 P1 |
| 337 | PI(18:2_18:2)-H | PI(36:4)-H | PI | (18:2_18:2) | (18:2) | (18:2) | 857.518557 | 857.51856 | 9.364 | C45 H78 O13 N0 P1 |
| 338 | PI(18:3_10:3)-H | PI(28:6)-H | PI | (18:3_10:3) | (18:3) | (10:3) | 741.362057 | 741.363259 | 6.29 | C37 H58 O13 N0 P1 |

| | | | | | | | | | | |
|---|---|---|---|---|---|---|---|---|---|---|
| 339 | PI(18:3_18:2)-H | PI(36:5)-H | PI | (18:3_18:2) | (18:3) | (18:2) | 855.502907 | 855.50291 | 8.691 | C45 H76 O13 N0 P1 |
| 340 | PI(18:3_18:3)-H | PI(36:6)-H | PI | (18:3_18:3) | (18:3) | (18:3) | 853.487257 | 853.48726 | 7.932 | C45 H74 O13 N0 P1 |
| 341 | PI(20:3e_10:0)-H | PI(30:3e)-H | PI | (20:3e_10:0) | (20:3e) | (10:0) | 761.461042 | 761.46104 | 1.538 | C39 H70 O12 N0 P1 |
| 342 | PI(20:3e_18:2)-H | PI(38:5e)-H | PI | (20:3e_18:2) | (20:3e) | (18:2) | 869.554942 | 869.55494 | 0.319 | C47 H82 O12 N0 P1 |
| 343 | PI(20:4e_12:1)-H | PI(32:5e)-H | PI | (20:4e_12:1) | (20:4e) | (12:1) | 785.461042 | 785.46104 | 4.684 | C41 H70 O12 N0 P1 |
| 344 | PI(21:4e)-H | PI(21:4e)-H | PI | (21:4e) | (21:4e) | | 633.304542 | 633.30454 | 3.169 | C30 H50 O12 N0 P1 |
| 345 | PI(22:0_14:1)-H | PI(36:1)-H | PI | (22:0_14:1) | (22:0) | (14:1) | 863.565507 | 863.56551 | 8.891 | C45 H84 O13 N0 P1 |
| 346 | PI(22:0_18:3)-H | PI(40:3)-H | PI | (22:0_18:3) | (22:0) | (18:3) | 915.596807 | 915.598604 | 7.781 | C49 H88 O13 N0 P1 |
| 347 | PI(22:1_18:2)-H | PI(40:3)-H | PI | (22:1_18:2) | (22:1) | (18:2) | 915.596807 | 915.605265 | 10.815 | C49 H88 O13 N0 P1 |
| 348 | PI(24:0_14:4)-H | PI(38:4)-H | PI | (24:0_14:4) | (24:0) | (14:4) | 885.549857 | 885.553158 | 12.791 | C47 H82 O13 N0 P1 |
| 349 | PI(24:1_18:2)-H | PI(42:3)-H | PI | (24:1_18:2) | (24:1) | (18:2) | 943.628107 | 943.62811 | 11.588 | C51 H92 O13 N0 P1 |
| 350 | PI(24:1_18:3)-H | PI(42:4)-H | PI | (24:1_18:3) | (24:1) | (18:3) | 941.612457 | 941.61246 | 11.045 | C51 H90 O13 N0 P1 |
| 351 | PI(28:0_18:3)-H | PI(46:3)-H | PI | (28:0_18:3) | (28:0) | (18:3) | 999.690707 | 999.69071 | 8.484 | C55 H100 O13 N0 P1 |
| 352 | PI(33:0_9:0)-H | PI(42:0)-H | PI | (33:0_9:0) | (33:0) | (9:0) | 949.675057 | 949.667967 | 4.108 | C51 H98 O13 N0 P1 |
| 353 | PI(36:1_10:3)-H | PI(46:4)-H | PI | (36:1_10:3) | (36:1) | (10:3) | 997.675057 | 997.67506 | 8.371 | C55 H98 O13 N0 P1 |
| 354 | PI(38:0_10:3)-H | PI(48:3)-H | PI | (38:0_10:3) | (38:0) | (10:3) | 1027.72201 | 1027.72201 | 9.285 | C57 H104 O13 N0 P1 |
| 355 | PI(38:4)-H | PI(38:4)-H | PI | (38:4) | (38:4) | | 885.549857 | 885.54986 | 11.309 | C47 H82 O13 N0 P1 |
| 356 | PI(38:5)-H | PI(38:5)-H | PI | (38:5) | (38:5) | | 883.534207 | 883.53421 | 11.353 | C47 H80 O13 N0 P1 |
| 357 | PI(38:5e)-H | PI(38:5e)-H | PI | (38:5e) | (38:5e) | | 869.554942 | 869.55494 | 13.316 | C47 H82 O12 N0 P1 |
| 358 | PI(40:2)-H | PI(40:2)-H | PI | (40:2) | (40:2) | | 917.612457 | 917.61246 | 11.439 | C49 H90 O13 N0 P1 |
| 359 | PI(42:2)-H | PI(42:2)-H | PI | (42:2) | (42:2) | | 945.643757 | 945.64376 | 4.16 | C51 H94 O13 N0 P1 |
| 360 | PI(42:4)-H | PI(42:4)-H | PI | (42:4) | (42:4) | | 941.612457 | 941.607132 | 4.179 | C51 H90 O13 N0 P1 |
| 361 | PI(48:5)-H | PI(48:5)-H | PI | (48:5) | (48:5) | | 1023.69071 | 1023.69104 | 7.883 | C57 H100 O13 N0 P1 |
| 362 | PI(51:1)-H | PI(51:1)-H | PI | (51:1) | (51:1) | | 1073.80026 | 1073.79589 | 13.066 | C60 H114 O13 N0 P1 |
| 363 | PI(51:6)-H | PI(51:6)-H | PI | (51:6) | (51:6) | | 1063.72201 | 1063.72622 | 5.227 | C60 H104 O13 N0 P1 |
| 364 | PIP(10:0e_16:0)-H | PIP(26:0e)-H | PIP | (10:0e_16:0) | (10:0e) | (16:0) | 791.411725 | 791.41172 | 10.442 | C35 H69 O15 N0 P2 |
| 365 | PIP(34:1_10:3)-H | PIP(44:4)-H | PIP | (34:1_10:3) | (34:1) | (10:3) | 1049.61009 | 1049.61009 | 4.737 | C53 H95 O16 N0 P2 |
| 366 | PIP(44:2)-H | PIP(44:2)-H | PIP | (44:2) | (44:2) | | 1053.64139 | 1053.64273 | 4.072 | C53 H99 O16 N0 P2 |
| 367 | PIP(6:0_10:3)-H | PIP(16:3)-H | PIP | (6:0_10:3) | (6:0) | (10:3) | 659.18754 | 659.187978 | 4.76 | C25 H41 O16 N0 P2 |

| | | | | | | | | | | |
|---|---|---|---|---|---|---|---|---|---|---|
| 368 | PIP2(4:0_15:0)-H | PIP2(19:0)-H | PIP2 | (4:0_15:0) | (4:0) | (15:0) | 787.247773 | 787.24777 | 4.786 | C28 H54 O19 N0 P3 |
| 369 | PMe(16:1_6:0)-H | PMe(22:1)-H | PMe | (16:1_6:0) | (16:1) | (6:0) | 519.309232 | 519.30923 | 4.258 | C26 H48 O8 N0 P1 |
| 370 | PMe(18:1e_16:0)-H | PMe(34:1e)-H | PMe | (18:1e_16:0) | (18:1e) | (16:0) | 673.517767 | 673.51777 | 11.251 | C38 H74 O7 N0 P1 |
| 371 | PMe(18:3_11:4)-H | PMe(29:7)-H | PMe | (18:3_11:4) | (18:3) | (11:4) | 605.324882 | 605.324486 | 6.545 | C33 H50 O8 N0 P1 |
| 372 | PMe(18:3)-H | PMe(18:3)-H | PMe | (18:3) | (18:3) | | 459.215332 | 459.21533 | 10.474 | C22 H36 O8 N0 P1 |
| 373 | PMe(20:2e_11:3)-H | PMe(31:5e)-H | PMe | (20:2e_11:3) | (20:2e) | (11:3) | 623.408217 | 623.40822 | 4.966 | C35 H60 O7 N0 P1 |
| 374 | PMe(20:3_11:4)-H | PMe(31:7)-H | PMe | (20:3_11:4) | (20:3) | (11:4) | 633.356182 | 633.351403 | 7.52 | C35 H54 O8 N0 P1 |
| 375 | PMe(29:0e)-H | PMe(29:0e)-H | PMe | (29:0e) | (29:0e) | | 605.455167 | 605.457358 | 5.033 | C33 H66 O7 N0 P1 |
| 376 | PMe(30:1)-H | PMe(30:1)-H | PMe | (30:1) | (30:1) | | 631.434432 | 631.434515 | 10.057 | C34 H64 O8 N0 P1 |
| 377 | PMe(32:3)-H | PMe(32:3)-H | PMe | (32:3) | (32:3) | | 655.434432 | 655.43443 | 9.493 | C36 H64 O8 N0 P1 |
| 378 | PMe(33:5)-H | PMe(33:5)-H | PMe | (33:5) | (33:5) | | 665.418782 | 665.41878 | 7.944 | C37 H62 O8 N0 P1 |
| 379 | PMe(41:4)-H | PMe(41:4)-H | PMe | (41:4) | (41:4) | | 779.559632 | 779.559505 | 3.601 | C45 H80 O8 N0 P1 |
| 380 | PS(30:1_10:3)-H | PS(40:4)-H | PS | (30:1_10:3) | (30:1) | (10:3) | 838.560361 | 838.56036 | 13.466 | C46 H81 O10 N1 P1 |
| 381 | PS(35:2)-H | PS(35:2)-H | PS | (35:2) | (35:2) | | 772.513411 | 772.513373 | 10.088 | C41 H75 O10 N1 P1 |
| 382 | PS(35:2e)-H | PS(35:2e)-H | PS | (35:2e) | (35:2e) | | 758.534146 | 758.53415 | 8.281 | C41 H77 O9 N1 P1 |
| 383 | SPHP(d15:0)-H | SPHP(d15:0)-H | SPHP | (d15:0) | (d15:0) | | 338.210186 | 338.21019 | 2.77 | C15 H33 O5 N1 P1 |
